# A scoping review of scientific concepts concerning motor recovery after stroke as employed in clinical trials

**DOI:** 10.3389/fneur.2023.1221656

**Published:** 2023-12-11

**Authors:** Martina Favetta, Alberto Romano, Nicola Valè, Blazej Cieslik, Sara Federico, Alessia Girolami, Deborah Mazzarotto, Giorgia Pregnolato, Anna Righetti, Silvia Salvalaggio, Enrico Castelli, Nicola Smania, Stefano Bargellesi, Pawel Kiper, Maurizio Petrarca

**Affiliations:** ^1^Movement Analysis and Robotics Laboratory (MARlab), Neurorehabilitation Unit, Neurological Science and Neurorehabilitation Area, Bambino Gesù Children's Hospital, IRCCS, Rome, Italy; ^2^Department of Health Systems Management, Ariel University, Ariel, Israel; ^3^Neuromotor and Cognitive Rehabilitation Research Center (CRRNC), Department of Neuroscience, Biomedicine and Movement Sciences, University of Verona, Verona, Italy; ^4^Laboratory of Healthcare Innovation Technology, IRCCS San Camillo Hospital, Venice, Italy; ^5^Spondilos Lab Centro Medico and Riabilitazione, Pordenone, Italy; ^6^Medicina Fisica e Riabilitazione, ULSS 4 Veneto Orientale, Jesolo, Italy; ^7^Laboratory of Computational Neuroimaging, IRCCS San Camillo Hospital, Venice, Italy; ^8^Padova Neuroscience Center, Università Degli Studi di Padova, Padua, Italy; ^9^Physical Medicine and Rehabilitation Unit, Azienda ULSS 3 Serenissima, Venezia, Italy

**Keywords:** motor control, motor learning, stroke, neurological rehabilitation, motor disorders, treatment outcome

## Abstract

The scientific literature on poststroke rehabilitation is remarkably vast. Over the last decades, dozens of rehabilitation approaches have been investigated. However, sometimes it is challenging to trace new experimental interventions back to some of the known models of motor control and sensorimotor learning. This scoping review aimed to investigate motor control models’ diffusion among the literature on motor recovery after stroke. We performed a literature search on Medline, Cochrane, Web of Science, Embase, and Scopus databases. The last search was conducted in September 2023. This scoping review included full-text articles published in English in peer-reviewed journals that provided rehabilitation interventions based on motor control or motor learning frameworks for at least one individual with stroke. For each study, we identified the theoretical framework the authors used to design the experimental treatment. To this aim, we used a previously proposed classification of the known models of motor control, dividing them into the following categories: neuroanatomy, robotics, self-organization, and ecological context. In total, 2,185 studies were originally considered in this scoping review. After the screening process, we included and analyzed 45 studies: 20 studies were randomized controlled trials, 12 were case series, 4 were case reports, 8 were observational longitudinal pilot studies, and 1 was an uncontrolled trial. Only 10 studies explicitly declared the reference theoretical model. Considering their classification, 21 studies referred to the robotics motor control model, 12 to the self-organization model, 8 to the neuroanatomy model, and 4 to the ecological model. Our results showed that most of the rehabilitative interventions purposed in stroke rehabilitation have no clear theoretical bases on motor control and motor learning models. We suggest this is an issue that deserves attention when designing new experimental interventions in stroke rehabilitation.

## 1 Introduction

Motor recovery after a stroke is a crucial aim in neurological rehabilitation. The incidence of stroke is estimated at over 13.7 million new cases per year globally (1). Motor impairment after a stroke can be related to different aspects of movement, such as control, learning, planning, and execution (2). Moreover, sensation deficits may affect motor control causing inaccurate feedback and affecting both motor planning and voluntary motor output (3). Although stroke is one of the most treated events in rehabilitation due to the long-term sequelae, there is no consensus on the optimal motor recovery strategy but only a consensus that physiotherapy is beneficial and that intensive repetitive and task-oriented training may foster neuroplasticity and maximize functional recovery (4, 5). However, although of paramount importance, this evidence recommends some treatment features but fails to help clinicians in selecting the most effective approaches and exercises.

Even when it comes to choosing the outcome measures to assess the effect of the rehabilitation, there is no shared definitive consensus. Recently, a Delphi study was conducted with this aim (6). From 119 assessment tools the authors found in the literature, they recommended a core set of nine to be used in clinical practice. Noteworthy, the authors underlined that this selection was meant to concern only clinical settings, and it was not useful to solve research issues in stroke motor rehabilitation (6).

Although recently there has been increasing interest in some crucial aspects of rehabilitation intervention such as the optimal feedback to be provided (7, 8), most of the interventions proposed in the last decades came from the pragmatic application of new evidence from different fields of knowledge. An example is the tremendous impact that neurophysiological advancements in the study of neural plasticity have had in the field of rehabilitation after stroke (3). A recent literature review highlighted the role of motor learning mechanisms, identifying clusters of principles and phenomena that play a key role in shaping recovery patterns in neurological diseases (9). Although this is an active field of study, it was suggested that some inertial factors may hamper its translation into clinical practice (10), resulting in the intervention being proposed more by the personal beliefs of the practitioners than driven by scientific hypothesis (10). The modest translation of scientific evidence into clinical settings also emerged from a recent review of the driven principles used during the design of robotic devices for neurological rehabilitation (11). Specifically, the authors suggested that often a theoretical reference was used to interpret the results a-posteriori, instead of being the theoretical background the research question was built on (11). In contrast, it would be expected that a theoretical frame drove the rehabilitative proposals and that the results of interventional studies could eventually be used to improve the reference theories.

In a neurorehabilitation framework, the concept of recovery may have different meanings. Following motor system damage, recovery could stem from a combination of innate biological processes and adaptive behavioral restitution or compensation (12). Behavioral restitution refers to the process of reverting to more typical patterns of motor control involving the affected effector (i.e., the body part interacting with the environment). Conversely, compensation denotes the patient’s capacity to achieve a goal by substituting a novel approach instead of relying on their pre-stroke behavioral patterns (13). For compensation processes, motor learning is required, and conversely, neural repair could not be necessary (14). In any case, the use of compensations can be maladaptive and acceptable only in severe deficits when there is very little chance of recovery.

These considerations support the importance of investigating the link between the physiological evidence on motor control and sensorimotor learning and the rehabilitation approaches that the literature has proposed in the past years. This topic is vast and complex since decades of scientific research have developed several scientific theories aiming to describe how humans control their movements (15–18). However, their impact on rehabilitation practice has been surprisingly overlooked. At the state of the art, there is no compelling evidence of the superiority of one theoretical framework over the others; instead, different models have been proven to effectively capture different aspects of human motor behavior (19–21). When dealing with this topic, the first issue is a taxonomy problem. Turvey and Fonseca, in a previous study, reviewed the existing motor control models and classified them into four categories considering if they are inspired by neuroanatomy, robotics, self-organization, or ecological realities (21). In the present review, we refer to this classification assuming it is comprehensive and suitable to be applied in stroke rehabilitation.

This scoping review aims to investigate motor control models’ diffusion among the literature on motor recovery after stroke. The review will examine the literature on motor rehabilitation after stroke searching for hypothesis-driven training.

This study will try to answer the following questions:

- Is motor rehabilitation after stroke driven by and based on scientific motor control and learning models?
- Are the models explicitly declared when experimental treatments are proposed in the literature?
- Can the interventions be classified even when there are no explicitly declared principles?

## 2 Materials and methods

The proposed scoping review was conducted in accordance with the JBI methodology for scoping review (22), and Preferred Reporting Items for Systematic reviews and Meta-Analyses extension for Scoping Reviews (PRISMA-ScR) (23) was adopted as a guideline for the reporting.

### 2.1 Eligibility criteria

To ensure that the review included studies relevant to motor rehabilitation interventions after stroke, we used the population-concept-context (PCC) framework. The eligibility criteria included patients with stroke and specific concepts related to motor control, motor learning, and theories/approaches. The criteria also included the context of motor rehabilitation, which ensured that the review only included studies that involved interventions aimed at improving motor function after stroke.

### 2.2 Type of source

This scoping review considered various experimental study designs, including randomized controlled trials, non-randomized controlled trials, case series, and individual case reports. Systematic reviews and meta-analyses were not included as they typically involve the synthesis of existing studies rather than original data collection. Additionally, text, opinion papers, and letters were not deemed appropriate or useful to meet the objectives of this scoping review as they do not typically involve empirical data collection or analysis.

### 2.3 Search strategy

The following bibliographic databases were searched: MEDLINE, Cochrane, Web of Science, Embase, and Scopus. The search strategies were drafted by an experienced researcher and further refined through the snowballing approach and team discussion. Searching terms were identified based on the selected PCC framework. Thus, we selected studies that involved neurological patients with strokes, and concept of motor control and motor learning theories/approaches, motor rehabilitation and related motor outcome. We also included additional search terms to ensure that we covered all relevant studies. The identified search terms were searched within the titles, abstracts, and keywords of the articles. The databases that allow controlled vocabularies (e.g., medical subject headings and Emtree) were searched with terms belonging to controlled and not controlled vocabularies. No limitation was applied for the publication year. The final search strategy for each searched database can be found in Supplementary Table 1. The last search was conducted in September 2023, including the articles published up to the end of August 2023.

**Figure 1:**
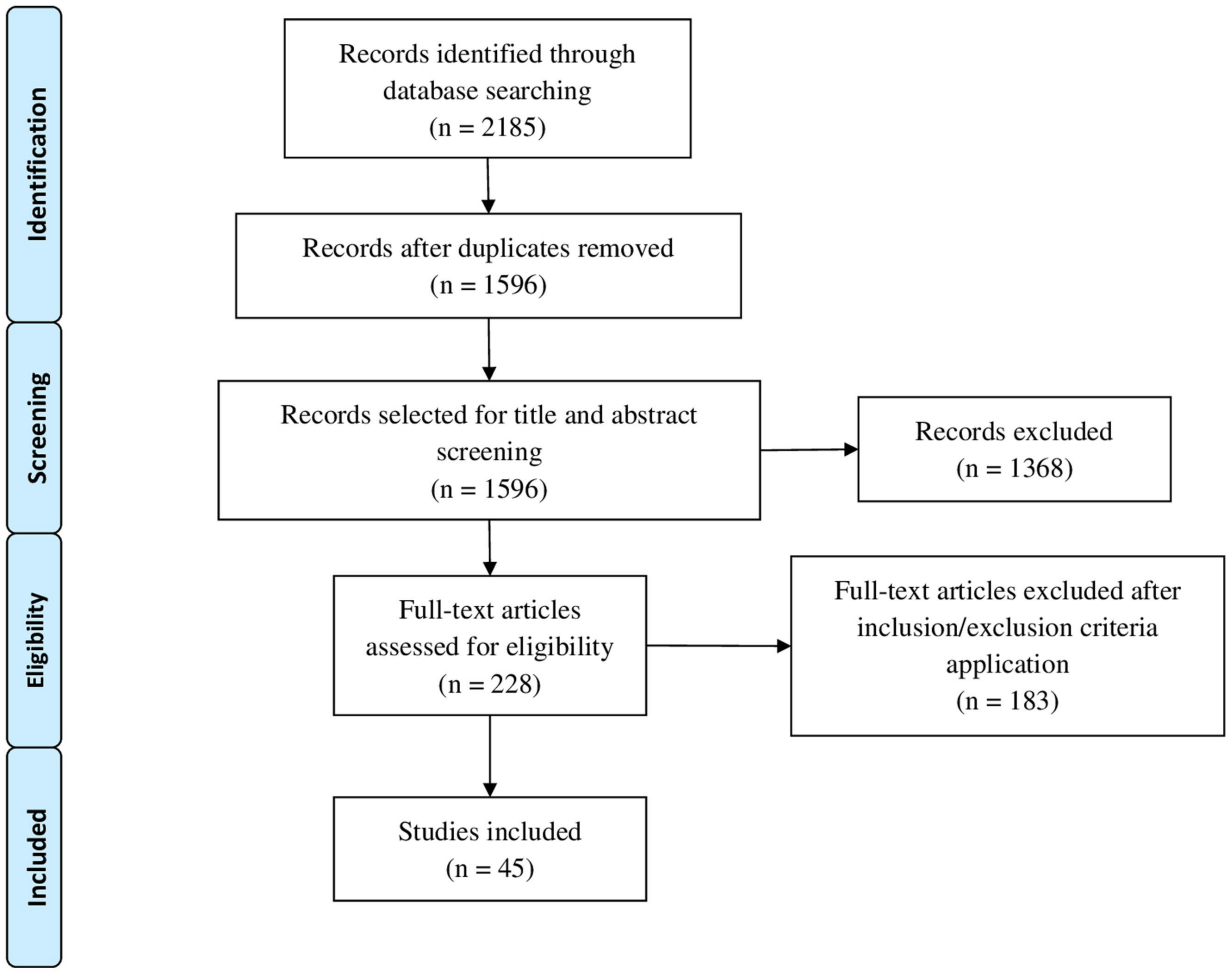
PRISMA flowchart of the study’s selection process.

Once retrieved, the database search results were exported into EndNote, and duplicates were removed. After removing duplicates, the title and abstract of each retrieved article were checked by three independent researchers, and articles related to stroke and motor control or motor learning were selected for the full-text read. Conflicts underwent group discussion until a consensus was reached. Articles selected for the full text were read and assessed by two independent researchers with a group discussion resolving any disagreements. This process ensured that we included only studies that met our inclusion criteria. The study selection process is depicted in Figure 1.

### 2.4 Inclusion criteria

This scoping review includes full-text articles published in English in peer-reviewed journals that present rehabilitation interventions based on motor control or motor learning principles for at least one individual with stroke.

### 2.5 Exclusion criteria

Articles not written in English, book chapters, review papers, article commentaries, conference abstracts or posters, and articles not included in the selected databases were excluded from this review. No exclusion criteria were applied for the study design to allow the identification of all the treatments based on motor control and motor learning principles.

### 2.6 Data extraction

From each included study, information concerning the study aims, the used outcome measures, and the declared motor control or motor learning theoretical frameworks were extracted. When a reference theoretical framework for the rehabilitation intervention was not clearly stated, it was reported as “not declared.” The reported bibliographic references related to the declared theoretical framework were also extracted. The findings from the included studies were synthesized through a narrative synthesis, presenting a comprehensive overview of the different motor rehabilitation interventions after stroke. The narrative synthesis helped to identify the scientific motor control and learning models that drive these interventions, the extent to which these models are explicitly declared, and how the interventions can be classified even when there are no declared principles.

For this reason, we categorized all included articles into four frameworks of motor control models in accordance with an influential study on this topic by Turvey and Fonseca (24). The authors suggested that motor control theories and models previously proposed may be classified according to their source of inspiration: neuroanatomy, robotics, self-organization, and ecological realities.

Models referring to the neuroanatomic category consider movement control essentially as a neural task. In this view, movement emerges as a combination of motor programs stored in the central nervous system (CNS) that selects and adapts them to perform a specific task. The subject acts as an executive system, and the cortical and spinal systems are used as a keyboard to play them. Neuromotor treatments based on this framework aim to rebuild motor programs lost after the brain lesion through the systematic repetition of specific movements. The learned motor programs are then assembled to explicitly construct the action.

Over the last decades, the development of robotics and the associated control systems inspired a series of human motor control theories that, in Turvey’s and Fonseca’s classification, fall into the category of robotics. These models suggest that the body is not a mere executor of neural commands. Instead, the physical properties of the limbs play a significant role in motor control. According to this perspective, when the CNS plans a movement, it explicitly computes the kinematics and dynamics of that movement, defining its trajectory and mapping through inverse kinetic and kinematics into motor torque and muscle activation. To do so, it can be hypothesized that the CNS shall have, at some level, knowledge of the physical laws involved in the body’s mechanics. Internal models allow the CNS to perform these computations through the prediction of movement‘s consequences (forward model) or the analytic definition of the motor command needed to perform a desired trajectory (inverse model) (16, 18). Noteworthy there is neurophysiological evidence that does not support the inverse model representation in the cerebellum (25).

Therefore, we included in this category neuromotor treatments that involve motor learning through external feedback chosen by the therapist (augmented feedback) and training that provides online feedback on the movement’s execution focusing explicitly on trajectories and applied forces. Augmented feedback can be delivered without providing explicit information, but this was not implemented in the articles included in our study. These treatments can also include physical constraints to movement (e.g., planar robots for upper limbs). These first two models suggest a hierarchical ordering of neuroanatomical structures and processes of control.

Another framework of motor control models included theories inspired by the self-organization concept. In this view, motor control emerges from and is influenced by the dynamic interaction between three systems: the CNS, the body, and the environment. In the context of motor learning, the system is left free to act and the only feedback that modulates learning is the achievement of the goal of the action. In this category, we included interventions that leave the system free to move until converging on a solution. In this perspective, the training of a neuromotor task has no constraints, the therapist does not manipulate the feedback, and the subject has more freedom of action during the execution of the exercise.

Recently, a series of theories proposed that movements and postures are controlled and coordinated to realize functionally specific acts based on the perception of affordances (26). The action is seen as intrinsically related to the environment and the context in which it is performed. The treatments that fall into this category (ecological) include a careful choice of the task to perform and the constraints to apply so that when the subject is engaged in the specific task, the desired motor behavior emerges from the biomechanical and informational constraints exchanged with the environment. Task and context become tools for bringing out motor behavior. The treatment is controlled by the therapist who prepares tasks and contexts to bring out the absent and/or desired motor action.

In the first model, the speculation was made that one can pinpoint both the anatomical regions being controlled and the origins of this control. In the progression from the first and last models, the ‘what’ and ‘where’ of control become increasingly less concrete and less expressible in anatomical terms.

To allocate studies into different categories, we referred to the background theoretical framework declared in the included studies. When this was not mentioned explicitly, we referred to the intervention used in the single studies following the criteria previously described. To highlight these different classification procedures, when reporting the results of this process in Supplementary Table 2, we divided between studies in which the theoretical framework was explicitly declared and studies in which it was not. For the studies in which the theoretical framework was explicitly declared, we evaluated the coherence between methods and the theoretical model declared. For all the included studies, we also analyzed the coherence between the aims and the outcome measures declared. For each of these two topics examined, we assigned a green dot if coherence was total and a yellow one if partial.

## 3 Results

Figure 1 shows the flowchart of the study selection process. Forty-five studies were eventually included in our review and analyzed. The synoptic table with the overview of the studies is available in Supplementary Table 3. From a methodological perspective, 20 (44.4%) were randomized controlled trials, 12 studies (26.7%) were case series, 4 (8.9%) were case reports, 8 (17.8%) were observational longitudinal pilot studies, and 1 (2.2%) was uncontrolled trial. The number of included patients varies between 50 (22) and 1 (26–29), with a greater prevalence of studies with more than 20 patients included (23) (51.1%). Only 1 study (a case report) refers to a child affected by hemiplegia poststroke (29) because childhood stroke is a rare event. From the intervention identification perspective, 27 studies had specific training for upper limb motor recovery, both considering reaching function and hand dexterity (30–32), 12 studies had lower limb and gait training, 3 studies had balance training, 2 studies had functional activities, and 1 study investigated the effect of PRISMA adaptation measure for recovery of spatial neglect (33).

As reported in Supplementary Tables 2, 3, most of the included studies (77.8%) did not explicitly mention a specific theoretical framework as the background of their proposed intervention (34). The robotics framework was most frequently used with 21 studies (46.7%) explicitly or implicitly basing their intervention on this model. Twelve studies (26.7%) referred to the self-organization framework, 8 (17.8%) to the neuroanatomy, and only 4 (8.9%) to the ecological one. Considering the studies that declared the theoretical framework they referred to, the coherence between the methods applied and the theoretical framework was good in 8 out of 10 studies (80.0%) and partial in 2 out of 10 studies (20.0%) (34, 35). Moreover, the coherence between the aims and the outcome measures was good in 42 out of 45 studies (93.3%) and partial in 3 out of 45 studies (6.7%) (28, 34, 36).

## 4 Discussion

This scoping review investigated whether and how rehabilitation interventions in patients with stroke motor sequelae were based on motor control and sensorimotor learning models. We included studies that explicitly or implicitly referred to theoretical frameworks of motor control and learning. Of the 1,596 records that entered the screening process, only 45 fulfilled the inclusion criteria and were included in the review. Of these, only 10 (22.2%) explicitly described the rationale of their proposed intervention referring to a specific motor control and learning model.

As for the studies that explicitly declared the theoretical framework they designed their treatment on, we found an overall good agreement between the declared framework and the proposed interventions. In two studies, a partial agreement was found. In detail, Dipietro et al. (35) designed a training based on robot-assisted pointing tasks to investigate whether improvement in accuracy and smoothness in such tasks resulted in improved smoothness in an untrained movement. The background described in the study conceived upper limb movements as built from the combination of simpler submovements (37). We reported partial coherence between the aim and reported rationale because if the authors assumed that upper limb movement could be conceived as a combination of simpler submovements, it is hard for the reader to understand how improvements in a specific task (i.e., robot-assisted pointing task) would generalize in a different task (i.e., circular movements). Reinkensmeyer et al. (34) proposed and evaluated a new model by comparing two groups performing therapeutic activities with and without a robotic device. However, the non-robotic-assisted treatment description was limited, preventing correctly understanding the highlighted motor learning components that were applied to it. Furthermore, although the new model could be categorized in the self-organization category (as it proposed that the learning occurs based on knowledge-of-result feedback and is linked neither to the level of assistance provided nor to the range and speed of practiced movement), the proposed training did not match the assumptions of this category. Indeed, the augmented feedback was provided during the robotic-assisted training, and the subject could not freely explore all the movement possibilities.

In light of the vast literature on rehabilitation in patients with stroke, the scant number of studies that explicitly declared their theoretical frameworks suggested that this information plays little role in describing the proposed intervention. In other words, there are several clinical rehabilitation trials in which it is difficult, if not impossible, to find any theoretical reference on the selected treatment. Noteworthy, this does not mean that in most interventional studies, the rationale of interventions is not declared but rather that the interventions are not reported to be designed on the known motor control and sensorimotor learning models. Although it can be seen as a minor issue, this is a crucial flaw that may significantly affect the quality of the research on neurorehabilitation.

First, this may foster the conduction of several clinical trials with limited clinical impact. Improving our understanding of crucial aspects associated with motor learning (e.g., optimal dose, principles of applications, and feedback manipulation) should be encouraged before focusing on the comparison of the effect of different interventions (e.g., when comparing experimental treatments to conventional physical therapy) (38). Designing experimental interventions based only on previous trials, without a clearly described theoretical background, makes it difficult to interpret and translate their results, eventually failing to improve the clinical practice. It is important to underline that the progression of knowledge in neurophysiology, especially on neuroplasticity mechanisms, allowed us to understand some characteristics that neurorehabilitation intervention should have to foster experience-dependent plasticity and functional motor recovery (3). As an example, there is a shared agreement that repetitive task-oriented and engaging practices may foster stroke patients’ recovery. Some of these principles helped to introduce some rehabilitation techniques that showed a strong level of evidence (e.g., constraint-induced movement therapy) (39). However, although all the rehabilitation interventions should follow these principles irrespective of the theoretical framework they are based on, we argue that it is the theoretical models of motor learning and motor control that inspire and define the actual design of exercises (e.g., choosing feedback modalities and adding perturbations or facilitations).

Second, and arguably most importantly, overlooking these aspects has led the literature to focus more on the device used than the treatment itself (11). This was particularly apparent in the context of robotic devices for rehabilitation. Indeed, when a new technology is ready and in fashion, it could happen that, without a clear scientific hypothesis, the development of new solutions might be confined to pragmatism and the personal feelings of practitioners (10).

In the current scoping review, a classification system for the theoretical models underlying the rehabilitation treatments for people with stroke was proposed based on the one presented by Fonseca et al. (24). This classification system could represent a valid tool for researchers in the field of motor rehabilitation, providing a reference to describe the foundation principles for rehabilitation treatment proposals. Moreover, the possibility to classify the treatments according to their theoretical framework (even when it is not explicitly declared) laid the basis for future meta-analyses to assess the efficacy of a theoretical approach instead of specific treatments. This opportunity gains value in light of the results of the proposed interventions: all the analyzed studies reported rehabilitative success and revealed their functional efficacy but also highlighted the lack of a valid and effective theoretical framework explaining the participants’ improvements after such different treatments. Identifying a theoretical framework appears mandatory for conducting further steps in the knowledge of the motor recovery process.

Among the four models proposed in the classification system, the most used was the robotics model. This model dues its name to the fact that it was inspired by the studies of control systems for robotic devices, and it was not surprising that most of these studies investigated robot-assisted training. The predominance of the robotics model in the current review reflects the wide impact that robotic devices have had on neurorehabilitation research. Indeed, robotics is one of the augmenting techniques aiming to exploit the enriched environment for providing augmented feedback, information, and repetitions to patients. The concept of augmented modalities involves the notion that enriching the external environment in which animals or subjects interact can result in significant modifications to their own functional systems both at a central level (e.g., CNS) and a peripheral level (e.g., muscles) (40, 41).

On the other hand, the least used reference model was the ecological framework. This model refers to selecting specific tasks and contexts to facilitate the emergence of the desired motor behavior. It is rarely considered because of the obvious difficulty in standardizing the interventions investigated in research in the rehabilitation field. Indeed, the ecological model imposes a high individualization of the selected tasks and contexts. Therefore, the same conditions can lead to different motor behaviors in different people, complicating the achievement of the replicability required in clinical trials.

Finally, the agreement between the objectives and outcome measures in the included studies was investigated and found adequate in 37 out of 40 studies (92.5%). This result highlighted the researchers’ attention to selecting appropriate measurements that answer their research questions. We did not find this agreement in three studies. The measurement used by Reinkensmeyer et al. (34) partially agreed with their study’s aim. The authors intended to prove the similar effect of robotic and non-robotic treatments. However, they assessed the outcome measures in a task that was a part of the robotic training, implying the participants enrolled in the robotic training could have been facilitated in the task performance. Furthermore, in the studies of Smedes and da Silva (28) and Tretriluxana et al. (36), the aims stated the intention to investigate the feasibility of the proposed treatments, but no feasibility measures were collected. This result was satisfactory as using adequate outcome measures was essential in producing high-quality research. However, when an intervention is aimed at activating motor learning processes, appropriate measures of motor learning are required to be collected. Although the in-depth analysis of the used outcome measures goes beyond the scope of the current review, a recent literature review suggested that selecting an adequate motor learning measure should be based on the treatment focus and that this approach is currently lacking (42).

This scoping review underlined that most of the interventions proposed in stroke motor rehabilitation research had no declared motor control or learning models as theoretical scientific frameworks. This aspect highlighted that, in stroke rehabilitation, the authors usually stress the efficacy of the proposed intervention more than the theoretical model used. The presentation of rehabilitation interventions not based on solid and explicit theoretical models could provide pragmatic procedures but is insufficient to understand the mechanism underlying the rehabilitation processes, restraining the growth of rehabilitation as a scientific discipline. Future rehabilitative trials should be driven by solid scientific hypotheses on motor control and learning principles to promote rehabilitation as a scientific-driven process.

## Author contributions

MF, ARo, NV, BC, SF, AG, DM, GP, ARi, SS, EC, NS, SB, PK, and MP contributed to devising and planning the study. MF, ARo, NV, BC, SF, AG, DM, GP, ARi, SS, PK, and MP screened abstracts, full texts, and contributed to the data extraction. MF, ARo, NV, PK, GP, and MP wrote the first draft of the study. SF, SS, MF, ARo, AG, GP, EC, NS, SB, and NV revised the manuscript. All authors contributed to the article and approved the submitted version.
