# Supplementary material for: A scoping review of scientific concepts concerning motor recovery after stroke as employed in clinical trials
Source: Front Neurol. 2023 Dec 11;14:1221656. doi: 10.3389/fneur.2023.1221656 (PMC10749504; doi:10.3389/fneur.2023.1221656)
Supplement: Supplementary file 1 [file Table_1.docx]

***Supplementary Material – Table1***

**A Scoping Review of Scientific Concepts Concerning Motor Recovery After Stroke as Employed in Clinical Trials**

**Martina Favetta, Alberto Romano, Nicola Valè, Blazej Cieslik, Sara Federico, Alessia Girolami, Deborah Mazzarotto, Giorgia Pregnolato*, Anna Righetti, Silvia Salvalaggio, Enrico Castelli, Nicola Smania, Stefano Bargellesi, Pawel Kiper and Maurizio Petrarca**

*** Correspondence**: Giorgia Pregnolato: [giorgia.pregnolato@hsancamillo.it](mailto:giorgia.pregnolato@hsancamillo.it)

**Table1.** Search strategy and number of retrieved results from each searched database.

| **Database** | **Search string** | **Results (number of articles)** |
| --- | --- | --- |
| Medline | ((("stroke"[Mesh]) OR "stroke"[tiab] OR "cerebrovascular accident"[tiab]) AND (("Motor control"[tiab] OR "Motion control"[tiab] OR "motor learning"[tiab] OR "motor skill* acquisition"[tiab]) AND ("principle*"[tiab] OR "theor*"[tiab] OR "framework*"[tiab] OR "conceptual model*"[tiab] OR "model*"[tiab] OR "hypothes*"[tiab] OR "knowledge translation"[tiab])) AND (("Rehabilitation"[Mesh]) OR ("Recovery of Function"[Mesh]) OR ("Therapeutics"[Mesh]) OR "Rehabilitation"[tiab] OR "Recovery of Function"[tiab] OR "Therapeutics"[tiab] OR "Habilitation"[tiab] OR "Restoration"[tiab] OR "Recovery"[tiab] OR "therap*"[tiab] OR "*therapy"[tiab] OR "intervention"[tiab] OR "treatment"[tiab])) AND ([tiab]) | 389 |
| Cochrane | ([mh stroke] OR "stroke" OR "cerebrovascular accident"):ti,ab,kw AND (("Motor control" OR "Motion control" OR "motor learning" OR (motor NEAR/3 skill* NEAR/3 acquisition)) NEAR/10 (principle* OR theor* OR framework* OR (conceptual NEAR/3 model*) OR model* OR hypothes* OR "knowledge translation")):ti,ab,kw AND ([mh Rehabilitation] OR [mh "Recovery of Function"] OR [mh Therapeutics] OR "Rehabilitation" OR "Recovery of Function" OR "Therapeutics" OR "Habilitation" OR "Restoration" OR "Recovery" OR therap* OR *therapy OR intervention* OR treatment*):ti,ab,kw | 97 |
| Web of Science | TS=(( "stroke" OR "cerebrovascular accident" ) AND (( "Motor control" OR "Motion control" OR "motor learning" OR ( motor NEAR/3 skill* NEAR/3 acquisition ) ) NEAR/10 ( principle* OR theor* OR framework* OR (conceptual NEAR/3 model*) OR model* OR hypothes* OR "knowledge translation" )) AND ( "Rehabilitation" OR "Recovery of Function" OR "Therapeutics" OR "Habilitation" OR "Restoration" OR "Recovery" OR therap* OR *therapy OR intervention* OR treatment* )) | 237 |
| Embase | ('cerebrovascular accident'/exp OR 'stroke':ab,ti OR 'cerebrovascular accident':ab,ti) AND ('motor control'/exp OR 'motor learning'/exp OR 'motor control':ab,ti OR 'motion control':ab,ti OR 'motor learning':ab,ti OR ('motor' NEAR/3 'skill*' NEAR/3 'acquisition')) AND ('theory'/exp OR ('conceptual' NEAR/3 'model') OR 'model'/exp OR 'principle*':ab,ti OR 'theor*':ab,ti OR 'framework*':ab,ti OR 'model*':ab,ti OR 'hypothes*':ab,ti OR 'knowledge translation':ab,ti) AND ('therapy'/exp OR 'rehabilitation':ab,ti OR 'recovery of function':ab,ti OR 'therapeutics':ab,ti OR 'habilitation':ab,ti OR 'restoration':ab,ti OR 'recovery':ab,ti OR 'therap*':ab,ti OR 'intervention*':ab,ti OR 'treatment*':ab,ti) | 1231 |
| Scopus | TITLE-ABS-KEY( ( "stroke" OR "cerebrovascular accident" ) AND (( "Motor control" OR "Motion control" OR "motor learning" OR ( motor W/3 skill* W/3 acquisition ) ) W/10 ( principle* OR theor* OR framework* OR (conceptual W/3 model*) OR model* OR hypothes* OR "knowledge translation" )) AND ( "Rehabilitation" OR "Recovery of Function" OR "Therapeutics" OR "Habilitation" OR "Restoration" OR "Recovery" OR therap* OR intervention* OR treatment* ) ) | 231 |
