# Supplementary material for: A scoping review of scientific concepts concerning motor recovery after stroke as employed in clinical trials
Source: Front Neurol. 2023 Dec 11;14:1221656. doi: 10.3389/fneur.2023.1221656 (PMC10749504; doi:10.3389/fneur.2023.1221656)
Supplement: Supplementary file 2 [file Table_2.docx]

***Supplementary Material – Table 2***

**A Scoping Review of Scientific Concepts Concerning Motor Recovery After Stroke as Employed in Clinical Trials**

**Martina Favetta, Alberto Romano, Nicola Valè, Blazej Cieslik, Sara Federico, Alessia Girolami, Deborah Mazzarotto, Giorgia Pregnolato*, Anna Righetti, Silvia Salvalaggio, Enrico Castelli, Nicola Smania, Stefano Bargellesi, Pawel Kiper and Maurizio Petrarca**

*** Correspondence**: Giorgia Pregnolato: [giorgia.pregnolato@hsancamillo.it](mailto:giorgia.pregnolato@hsancamillo.it)

**Table2.** The classification of the theoretical frameworks of the studies.

| **Reference** | **Declared theoretical framework** | **Reported references to theoretical framework** | **Treatment frameworks of motor control** | **Coherence between methods and theoretical model** | **Aim** | **Outcome measures** | **Coherence between aims and outcome measures** |
| --- | --- | --- | --- | --- | --- | --- | --- |
| **Declared** |  |  |  |  |  |  |  |
| Dipietro, L., et al. (2009) (1) | Human movements are generated by discrete building blocks of movement, often labeled submovements, which represent an ‘alphabet’ of primitive movements. Recovery seems to progress first by rapidly reacquiring the ‘‘alphabet’’, and then, over a longer time, by reacquiring the means to combine these elements into ‘‘words’’ or ‘‘phrases’’. | Duarte, J.E., et al. (2013) (2)*.* | Neuroanatomy | 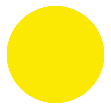 | To explore whether untrained UL movements during motor recovery from stroke exhibit changes in smoothness and sub-movements, thereby investigating their nature. | - FMA-UE - Circle drawing quality | 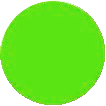 |
| Grimm, F., et al, (2016) (3) | Information on movement quality has therefore been incorporated as implicit closed-loop feedback in the virtual environment of an exoskeleton-based rehabilitation device. Specifically, the continuous visual feedback of the whole arm kinematics allowed the patients to adjust their movement quality online during each task; an approach closely resembling natural motor learning | Grimm, F., et al. (2016) (4) | Robotics | 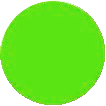 | To assess closed-loop task difficulty adaptation during VR reach-to-grasp UL training assisted with an exoskeleton in stroke rehabilitation. | - UL accuracy, temporal efficiency, ROM  -FMA-UE | 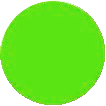 |
| Grimm, F., et al. (2016) (4) | The three-dimensional visualization of the arm was applied during each task as an implicit online feedback of movement quality, since explicit information can disrupt motor learning in stroke patients. In the present feasibility study, we extended this line of research by incorporating information on movement quality as implicit closed-loop feedback in the virtual environment of an exoskeleton-based rehabilitation device suitable for severely affected stroke patients who require gravity-support to perform activities of daily living such as reach-to-grasp exercises.  an approach closely resembling natural motor learning. | Boyd, L.A., and Winstein, C.J. (2004) (5) | Robotics | 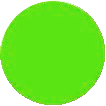 | To test feasibility and clinical validity of VR visualization and closed-loop feedback of joint-specific movement in severely stroke patients.  To quantify the individual degree of UL natural movement restoration or compensation. | - FMA-UE  - UL ROM | 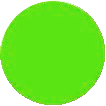 |
| Longo, D., et al. (2022) (6) | The Muscle Shortening Maneuver (MSM), a physical therapy approach, was introduced by Grimaldi et al. in the eighties and is derived from Feldman’s λ model of motor control  According to the model, the nervous system is able to modify the equilibrium state of the neuromuscular system by changing its parameters, thus controlling movement | Melchiorre D., et al. (2014) (7);  Feldman, A.G. (1998) (8) | Neuroanatomy | 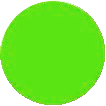 | To investigate the hypothesis that MSM could influence the modulation of the TSRT.  To investigate the MSM as a rehabilitation technique to improve body functions and activities in individuals with limitations due to chronic stroke. | Montreal Spasticity Measure Device (EMG and goniometer) | 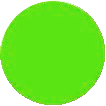 |
| Petrarca, M., et al. (2011) (9) | High-intensity and task specific program of strengthening exercises and training and functional therapy resulted in improved strength and functional performance maintained over time. Motor learning principle based on Feldman’s Equilibrium point model. Ecological approach. | Feldman, A.G. (1986)  (10);  Pierro, M.M., et al. (2005) (11) | Ecological | 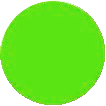 | To move in the direction of a person-oriented decision-making process on gait rehabilitation by analyzing the recovery from stroke of a six-year-old boy over three years. | -kinetics and kinematics (Gait Analysis)  -GMFM  -PEDI | 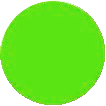 |
| Pollock, C.L., et al. (2014) (12) | Challenge Point Framework (CPF) Model;extrinsic feedback (visual feedback); knowledge of results (KR) and knowledge of performance (KP) | Guadagnoli, M.A., et al. (2004) (13) | Ecological | 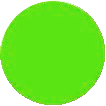 | To retrain of multidirectional stepping reactions may be informed by the CPF to improve balance function in people with stroke. | - Walking balance Community Balance and Mobility Scale  - ABC scale  - Kinematics (Gait analysis) | 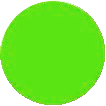 |
| Reinkensmeyer, D.J., et al. (2009) (14) | New use-dependent brain organization model:   - The model focuses on strokes that partially destroy the corticospinal system;   - The authors assume that motor control gains are caused by improvements in the ability to activate the spared portions of the corticospinal tract that activate motor neuron pools;   - they further assume that the motor system learns how to better activate spared corticospinal tracts by searching for optimal activation patterns using a stochastic local search process. The teaching signal that guides this search is a scalar measure of the movement success caused by the current activation pattern relative to the most successful, previously-tried activation pattern;   - the action of trying to move accounted for the practice-dependent arm movement recovery, independently of the specific ROM achieved or the level of assistance.   - it is the action of trying to move that automatically and inherently elicits the teaching signal required for the neural reorganization supporting the movement recovery, independently of the quantitative task feedback.  - The range and speed of the practiced movements do not alter the rate of recovery; all that matters is the magnitude of the teaching signal (in this case, peak force) achieved by the current pattern of activation. | Anderson, R.W. (1993) (15);  Williams R.J. (1992) (16);  Werfel, J., et al. (2005) (17);  Mazzoni, P., et al. (1991) (18);  Maier, M.A., et al. (1998) (19);  Fetz, E.E., et al. (1989) (20);  Ashe, J., (1997) (21) | Self-organization | 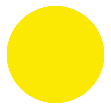 | To demonstrate that different dose-matched, UL rehabilitation training techniques can result in similar improvement in movement ability after stroke. | - strength (shoulder flexion/elbow extension)  - movement speed  - movement coordination | 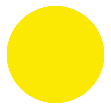 |
| Rowe, J.B., et al. (2017) (22) | Success is known to encourage motivation, self-efficacy, and willingness to practice. Active assistance increases success, and therefore presumably activates these positive learning features. Alternately, active assistance might promote Hebbian plasticity by increasing the amount of proprioceptive input in a way that is time-correlated with attempted motor activity. | Duarte J.E., et al. (2013) (2);  Goodman, R.N., et al. (2014) (23);  Abe, M., et al. (2011) (24);  Reinkensmeyer D.J. (2003) (25) | Robotics | 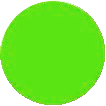 | To determine the therapeutic effects of high and low levels of robotic assistance during finger training | - BBT  - NHPT  - NIHSS  - FMA-UE  - ARAT  - Lateral Pinch Strength Test  - Motivation  - self efficacy | 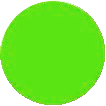 |
| Shaphe, A., et al. (2018) (26) | In a natural closed loop feedback control system, the physical motion of the body generates the visual cue in response to ambulation and in the absence of movement these cues are not generated. Visual inputs are most import external sensory cues regulating walking. | Goodman, R.N., et al. (2014) (23) | Robotics | 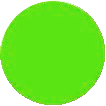 | To investigate the efficacy closed loop visual cues incorporated augmented VR environment on functional gait and community ambulation in stroke patients. | - Gait Analysis  - SF-SIS | 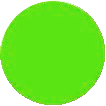 |
| Vilimovsky, T., et al. (2021) (27) | PAT (prism adaptation treatment) was delivered, by a physiotherapist, using the treatment protocol and equipment of the  Kessler Foundation Prism Adaptation Treatment (KF-PAT) |  | Ecological | 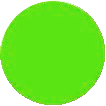 | PAT reduced visuospatial symptoms of spatial neglect among patients in an inpatient setting providing intensive rehabilitation care.  PAT enhanced the recovery of spatial neglect. | - CBS  - Bells test  - Line bisection  - Scene copying test | 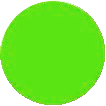 |
| **Not Declared** |  |  |  |  |  |  |  |
| De Bruyn, N., et al. (2021) (28) | N.D. |  | Neuroanatomy |  | To investigate differences in therapy-induced resting-state functional connectivity changes between additional sensorimotor therapy compared with motor therapy in the early-phase post stroke | - fMRI | 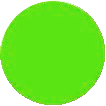 |
| Doost, M.Y., et al. (2021) (29) | N.D. |  | Ecological |  | To investigate whether training under the robotic active-assisted mode improves bimanual motor skill learning more than training under the active mode in stroke patients. | - Speed  -accuracy | 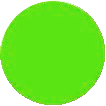 |
| García-Ramos, B. R., et al. (2023) (30) | N.D. |  | Self-organization |  | To explore the effect of a new game-based ocular virtual reality training on the cerebral activity in sensorimotor regions and accuracy in eye and hand movements in three different profiles of stroke survivors. | - FMA-UE;  - mean absolute error during the continuous tracking of a target task mentioned above;  - fMRI scanning during continuous tracking of a target tasks through eye-tranking and a hand-controlled joystick. | 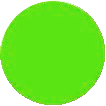 |
| Gilmore, P.E., & Spaulding, S.J. (2007) (31) | N.D. |  | Self-organization |  | To determine the effectiveness of combining a program of VOT and OT in learning the skill of donning socks and shoes poststroke | -KB-ADL socks and shoes subtests.  - COPM | 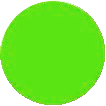 |
| Hegazy E. M. et al. (2022) (32) | N.D. |  | Self-organization |  | To compare the effect of a virtual reality training program and a task-oriented training program on the paretic upper limb function after stroke. | -UEFI  -grip strength test (using a dynamometer) | 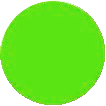 |
| Huang, C.Y., et al. (2022) (33) | N.D. |  | Self-organization |  | To identify the effects of immersive VR training on inflammation, oxidative stress, neuroplasticity and UL motor function in stroke patients. | -FMA-UE  -AROM (shoulder flexion, elbow extension, wrist extension, forearm supination and pronation)  -SSQ  -RPE molecular biomarkers (BDNF) | 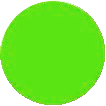 |
| Jonsdottir, J., et al. (2010) (34) | N.D. |  | Robotics |  | To examine the efficiency of EMG BFB training combined with theories of motor learning in improving performance and learning of gait parameters after stroke. | -Gait analysis | 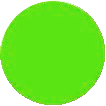 |
| Jonsdottir, J., et a.l. (2007) (35) | N.D. |  | Robotics |  | To evaluate the efficacy of task-oriented EMG-BFB in increasing push off power of the plantar flexors muscle on the affected side and to increase gait velocity in a population with hemiparetic stroke. | -Ankle power peak (W/kg)  -Velocity  - Stride length  -Knee flexion peak (degrees) | 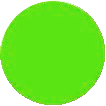 |
| Junata, M., et al. (2021) (36) | N.D. |  | Self-organization |  | To evaluate the effectiveness of the interactive RMT and CBT on chronic stroke survivors overall balance recovery reaction. | -BBS  -TUG  -FMA-UE  -Barthel index  -EMG activation | 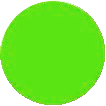 |
| Kamatchi K. et al. (2023) (37) | N.D. |  | Robotics |  | To assess the effectiveness of virtual reality in the rehabilitation of persons with stroke to improve the upper extremity function. | - FMA-UE  - sEMG signal amplitude | 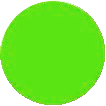 |
| Kim H., et al. (2023) (38) | N.D. |  | Robotics |  | To i evaluate the effectiveness of the of mirror therapy with video augmented wearable refection device on reach-to-grasp motor control and upper extremity motor function. | -Trunk kinematics  -FMA-UE  -MFT  -BBT | 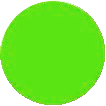 |
| Kim, G.J., & Chen, P. (2020) (39) | N.D. |  | Robotics |  | To examine the effects of instruction adherence on UL motor outcomes after highly structured intervention. | -MCQ -FMA-UE -WMFT  -FAS | 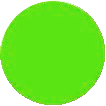 |
| Krishnamoorthy, V., et al. (2008) (40) | N.D. |  | Robotics |  | To describe the findings of training with a specially designed gravity-balanced orthosis, treadmill walking, FES, and principles of motor learning. | -TUG  -FMA-LE  -BBS  -Gait analysis | 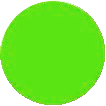 |
| Longatelli, V., et al. (2021) (41) | N.D. |  | Neuroanatomy |  | To investigate the gait rehabilitation process and evaluates motor re-learning in patients with subacute post-stroke by analysing and comparing LL muscular activation patterns. | - Gait functionality by means of clinical scales combined to obtain a Capacity Score (5-item modified Barthel index, MI, TMWT, 6MWT, FAC, TCT).  - Gait neuromuscular lower limbs pattern using surface EMG signals | 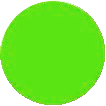 |
| Luque-Moreno, C., et al. (2019) (42) | N.D. |  | Self-organization |  | To determine whether a program that includes the combination of TR and RFVE decreases the level of spasticity of the PF muscles and improved gait function | - MAS  - FAC  - FIM | 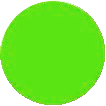 |
| Maenza, C., et al. (2021) (43) | N.D. |  | Robotics |  | To assess whether a rehabilitation approach focused on remediation of ipsilesional arm motor deficits in stroke survivors with moderate to severe contralesional arm paresis and with significant ipsilesional arm coordination deficits, improves functional performance and independence. | - JTHFT  - FIM  - Grip Strength hand dynamometer  - FMA-UE | 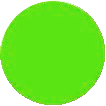 |
| Maggio, M.G., et al. (2021) (44) | N.D. |  | Neuroanatomy |  | To evaluate the usefulness of a RAGT equipped with augmented visuomotor feedback in improving LL sensorimotor function, gait performance and body representation.  To understand the putative neurophysiological correlates (EEG analysis) of the correlation between BR recovery and motor performance improvement. | - BES  - BUT  - FMA-LE  - FAB  - MoCA  - BDI  - SF12  - EEG | 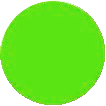 |
| Mazzoleni, S., et al. (2019) (45) | N.D. |  | Robotics |  | To investigate the effectiveness of combining tDCS and wrist robot-assisted rehabilitation in subacute stroke patients in comparison with the wrist robotic training only | - FMA-UE  - MAS (wrist muscles)  - MI  - BBT  - Kinematic parameters of wrist movements: abduction, adduction, extension, flexion | 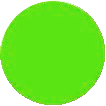 |
| Castro-Medina, K.G. (2023) (46) | N.D. |  | Robotics |  | To determine the effect of visual feedback on gait speed after stroke in adults with subacute and chronic stages. | - TMWT | 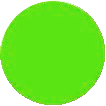 |
| Paolucci, T., et al. (2021) (47) | N.D. |  | Robotics |  | To determine the effects of an integrated rehabilitation protocol, including botulinum toxin and conventional rehabilitation exercise plus end-effector robotic training for UL functional recovery compared to training with the robot alone in post-chronic stroke | - FMA-UE  - MI  - MAS  - Numeric rating scale.  – BBT  - Frenchay Arm Test  - Barthel Index | 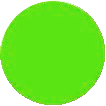 |
| Park, M., et al. (2019) (48) | N.D. |  | Robotics |  | To assess the clinical effectiveness of the VR-based rehabilitation device (Rapael Smart Board™) for the UL rehabilitation in chronic stroke patients.  To investigate the correlations between kinematic data from the Rapael Smart Board™ and clinical outcome. | - FMA-UE  - WMFT  - AROM (proximal upper extremities)  - Barthel index  - SIS | 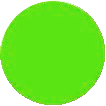 |
| Piron, L., et al. (2010) (49) | N.D. |  | Self-organization |  | To determine if a rehabilitation technique that aimed to augment the possibility of motor learning using VR (RFVE) could improve motor outcome scores significantly more than conventional treatment.  To compare the effects of a VR based technique with a control intervention of progressive therapy for the affected UL. | - FMA-UE  - ROM (Shoulder, Elbow, Wrist)  - AROM  - PROM  - MAS  -CAHAI  - CHART  - EMG analysis | 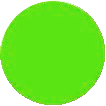 |
| Powers, J., et al. (2022) (50) | N.D. |  | Robotics |  | To investigate the effects of augmented feedback during overground gait training, on TGA | - NIHSS  - MCA  - TGA  - gait speed | 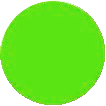 |
| Pundik, S., et al. (2022) (51) | N.D. |  | Neuroanatomy |  | To evaluate MyoPro as a tool for motor learning-based therapy for individuals with chronic UL impairment. | - FMA-UE; Shoulder, Elbow, Wrist  - PROM/AROM  - MAS  - CAHAI  - CHART  - OPUSsat | 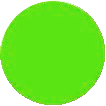 |
| Sainburg, R.L., et al. (2016) (52) | N.D. |  | Robotics |  | To test intense non-paretic arm training in improving motor coordination and functional performance in the trained arm, but also in improving functional independence and paretic arm function. | - FIM  - FMA-UE  - JTHFT | 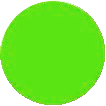 |
| Salameh, A., et al. (2022) (53) | N.D. |  | Self-organization |  | To develop and test a combination protocol of simultaneous brain stimulation and focused stance phase training for people with chronic stroke. | - TMWT  - TUG  - Functional Gait Assessment | 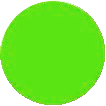 |
| Saleh, S., et al. (2017) (54) | N.D. |  | Self-organization |  | To compare the effect of robot-assisted VR repetitive task practice-based interventions on neural pattern reorganization. | - fMRI  - JTHFT | 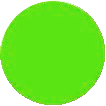 |
| Schreiber, J., et al. (2001) (55) | N.D. |  | Neuroanatomy |  | To determine the relationship between type of task and type of environment on retention and transfer of motor skills when applied to stroke survivors, as measured by time to complete the task and the number of errors. | - Number of errors in keyboard typing. | 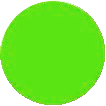 |
| Smedes F., & da Silva, L.G. (2019) (56) | N.D. |  | Neuroanatomy |  | To illustrate the clinical reasoning and the feasibility of applying PNF-concept as an alternative approach in patients who are not accepted or not suitable for CIMT. | - AROM/PROM wrist extension  - grip strength (handheld dynamometer)  - MAS  - FAT  - NHPT | 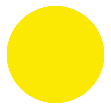 |
| Tretriluxana, J., et al. (2013) (57) | N.D. |  | Self-organization |  | To investigate the feasibility of Accelerated Skill Acquisition Program delivered during the 1- to 3-month outpatient interval in stroke survivors and included an assessment of Reach-to-Grasp coordination. | - Reach-to-Grasp  - WMFT  - SIS | 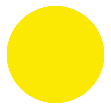 |
| Tsaih, P.L., et al. (2018) (58) | N.D. |  | Robotics |  | To determine the effects of constant force or variable force practice with task related EMGBFB-assisted exercise training on the TA muscle strength, balance, and LL motor function in people with chronic stroke. | - TA strength  - dynamic posturography  - walking speed  - TUG  - 6MWT | 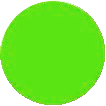 |
| Turolla, A., et al. (2013) (59) | N.D. |  | Robotics |  | To evaluate the efficacy of the proposed technological solution for the rehabilitation of hand and fingers motor function in poststroke patients | - FMA-UE  - NHPT  - speed  - smoothness (Jerk)  - fMRI | 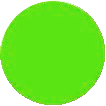 |
| Van Vugt, F.T., et al. (2016) (60) | N.D. |  | Robotics |  | To test the hypothesis whether rehabilitation benefits in music-supported therapy are due to auditory feedback-based motor learning. | - Barthel index  - NHPT  - Finger tapping measurements  - Auditory and auditory-motor tests | 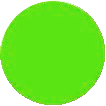 |
| Winstein, C., et al. (2019) (61) | N.D. |  | Self-organization |  | To explore the dose response and test the dosage of task-specific practice needed to achieve meaningful improvement in arm and hand use in chronic stroke survivors. | - MAL  - WMFT | 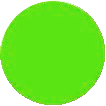 |
| Zollo, L., et al. (2011) (62) | N.D. |  | Robotics |  | To provide quantitative measure of biomechanical and motion planning features of arm motor control following upper-limb robot-aided motor therapy. | - FMA-UE  - Motor Power | 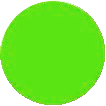 |

**Abbreviation list:** UL= Upper Limb; FMA-UE= Fugl-Meyer Assessment–Upper Extremity; VR= Virtual Reality; ROM= range of motion; MSM= Muscle Shortening Maneuver; TSRT=Tonic Stretch Reflex Threshold; EMG= electromyography; GMFM= Gross Motor Function Measure; PEDI= Pediatric Evaluation of Disability Inventory; CFP= Challenge Point Framework; KR= knowledge of results; KP= knowledge of performance; ABC=Activities-speciﬁc Balance Conﬁdence Scale; BBT= Box and Blocks Test; NHPT= Nine Hole Pegboard Test ; NIHSS= NIH Stroke Scale; ARAT= Action research arm test; SF-SIS= stroke impact scale; PAT= Prisma Adaptation Treatment; CBS= Catherine Bergego Scale; N.D.= Not Defined; fMRI= functional Magnetic Resonance Imaging; VOT= videotape feedback occupational therapy; OT= occupational therapy; KB-ADL=Klein Bell Activities of Daily Living Scale; COPM=Canadian Occupational Performance Measure; UEFI= Upper Extremity Functional Index test; AROM= range of active motion; SSQ= Simulator Sickness Questionnaire; RPE= Borg Scale of Perceived Exertion; BDNF= Brain-derived neurotrophic factor; BFB= biofeedback; RMT= Rapid Movement Training; CBT= Conventional Balance Training; BBS= Berg Balance Scale; TUG= Timed Up-and-Go test; MCQ= Manipulation Check Questionnaire; WMFT= Wolf Motor Function Test; FAS=Functional Ability Scale; FES= Functional Electrical Stimulation; MI= Motricity Index; TMWT= ten meters walking test; 6MWT= six minutes walking test; FAC= Functional Ambulatory Category; TCT= Trunk Control Test; TR= traditional rehabilitation; RFVE= reinforced feedback virtual environment; PF= plantar flex; MAS= Modified Ashworth Scale; FIM= Functional Independence Measure; JTHFT= Jebsen Taylor Hand Function Test; RAGT= robot-aided gait training; LL= Lower Limb; EEG= electroencephalogram; BR= body representation; BES=Body Esteem Scale; BUT= Body Uneasiness Test; FMA-LE= Fugl-Meyer Assessment–Lower Extremity; FAB= frontal assessment battery; MoCA= Montreal Cognitive Assessment; BDI= Beck depression inventory; SF12= Short Form-12 health status questionnaire; tDCS= Transcranial direct current stimulation; SIS= Stroke Impact Scale; PROM= Passive ROM; CAHAI= Chedoke Arm and Hand Activity Inventory; CHART= Craig Handicap Assessment and Rehabilitation Technique; MCA= Chedoke McMaster Stroke Assessment; TGA= Temporal Gait Asymmetry; OPUSsat= Orthotic and Prosthetic User’s Survey Satisfaction module; PNF= proprioceptive Neuromuscular Facilitation; CIMT= Constraint Induced Movement Therapy; FAT= Frenchay Arm Test; TA= Tibial Anterior; BART= Bilateral Arm Reaching test.

REFERENCES

1. Dipietro, L., Krebs, H.I., Fasoli, S.E., Volpe, B.T., Hogan, N. Submovement changes characterize generalization of motor recovery after stroke. Cortex. (2009) 45:318–24. doi: 10.1016/j.cortex.2008.02.008
2. Duarte, J.E., Gebrekristos, B., Perez, S., Rowe, J.B., Sharp, K., Reinkensmeyer, D.J. Effort, performance, and motivation: Insights from robot-assisted training of human golf putting and rat grip strength. In: 2013 IEEE 13th International Conference on Rehabilitation Robotics (ICORR). IEEE (2013). p. 1–6. doi: 10.1109/ICORR.2013.6650461.
3. Grimm, F., Naros, G., Gharabaghi, A. Closed-Loop Task Difficulty Adaptation during Virtual Reality Reach-to-Grasp Training Assisted with an Exoskeleton for Stroke Rehabilitation. Front Neurosci (2016) 15;10. doi: 10.3389/fnins.2016.00518.
4. Grimm, F., Naros, G., Gharabaghi, A. Compensation or restoration: Closed-loop feedback of movement quality for assisted reach-to-grasp exercises with a multi-joint arm exoskeleton. Front Neurosci (2016) 10:280. doi: 10.3389/fnins.2016.00280.
5. Boyd, L.A., Winstein, C.J. Providing Explicit Information Disrupts Implicit Motor Learning After Basal Ganglia Stroke. Learning & Memory (2004) 11(4):388–96. doi: 10.1101/lm.80104.
6. Longo, D., Santini, G., Cherubini, G., Melchiorre, D., Ferrarello, F., Bagni, M.A. The muscle shortening maneuver in individuals with stroke: a consideration-of-concept randomized pilot trial. Top Stroke Rehabil (2022) 1–13. doi: 10.1080/10749357.2022.2145741.
7. Melchiorre, D., Maresca, M., Bracci, R., Ravaschio, A., Valiensi, B., Casale, R., et al. Muscle shortening manoeuvre reduces pain and functional impairment in shoulder impingement syndrome: clinical and ultrasonographic evidence. Clinical and Experimental Rheumatology (2013).
8. Feldman, A.G. Spatial frames of reference for motor control. In: Progress in motor control, Vol 1: Bernstein’s traditions in movement studies. Champaign, IL, US: Human Kinetics (1998). p. 289–313.
9. Petrarca, M., Rossi, S., Bollea, L., Cappa, P., Castelli, E. Patient-centered rehabilitation, three years of gait recovery in a child affected by hemiplegia: case report. Eur J Phys Rehabil Med (2011) 47(1):35-47.
10. Feldman, A.G. Once More on the Equilibrium-Point Hypothesis (λ Model) for Motor Control. J Mot Behav (1986) 18(1):17–54. doi: 10.1080/00222895.1986.10735369.
11. Pierro, M.M., Bollea, L., Di Rosa, G., Gisondi, A., Cassarino, P., Giannarelli, P., et al. Anoxic brain injury following near-drowning in children. Rehabilitation outcome: Three case reports. Brain Inj (2005) 19(13):1147–55. doi: 10.1080/02699050500149973.
12. Pollock, C.L., Boyd, L.A., Hunt, M.A., Garland, S.J. Use of the Challenge Point Framework to Guide Motor Learning of Stepping Reactions for Improved Balance Control in People With Stroke: A Case Series Background and Purpose. Stepping reactions are important for walking (2014). doi: 10.2522/ptj.20130046.
13. Guadagnoli, M.A., Lee, T.D. Challenge Point: A Framework for Conceptualizing the Effects of Various Practice Conditions in Motor Learning. J Mot Behav (2004) 36(2):212–24. doi: 10.3200/JMBR.36.2.212-224.
14. Reinkensmeyer, D.J., Maier, M.A., Guigon, E., Chan, V., Akoner, O.M., Wolbrecht, E.T., et al. Do robotic and non-robotic arm movement training drive motor recovery after stroke, by a common neural mechanism? experimental evidence and a computational model. In: 2009 Annual International Conference of the IEEE Engineering in Medicine and Biology Society. IEEE (2009). p. 2439–41. doi: 10.1109/IEMBS.2009.5335353.
15. Anderson, R.W. Biased Random-Walk Learning: A Neurobiological Correlate to Trial-and-Error. Neural Networks and Pattern Recognition (1993) 221–44. doi: 10.1016/B978-012526420-4/50008-2.
16. Williams, R.J. Simple Statistical Gradient-Following Algorithms for Connectionist Reinforcement Learning. Vol. 8. (1992).
17. Werfel, J., Xie, X., Seung, H.S. Learning Curves for Stochastic Gradient Descent in Linear Feedforward Networks. Neural Comput (2005) 17(12):2699–718. doi: 10.1162/089976605774320539.
18. Mazzoni, P., Andersen, R.A., Jordan, M.I. A more biologically plausible learning rule for neural networks Proc Natl Acad Sci U S A. (1991) 88(10):4433-7. doi: 10.1073/pnas.88.10.4433.
19. Maier, M.A., Perlmutter, S.I., Fetz, E.E. Response Patterns and Force Relations of Monkey Spinal Interneurons During Active Wrist Movement. J Neurophysiol (1998) 80(5):2495–513. doi: 10.1152/jn.1998.80.5.2495.
20. Fetz, E.E., Cheney, P.D., Mewes, K., Palmer, S. Chapter 36 Control of forelimb muscle activity by populations of corticomotoneuronal and rubromotoneuronal cells. (1989). p. 437–49. doi: 10.1016/s0079-6123(08)62241-4.
21. Ashe, J. Erratum to “Force and the motor cortex.” Behavioural Brain Research (1997) 87(2):255–69. doi: https://doi.org/10.1016/S0166-4328(97)00752-3.
22. Rowe, J.B., Chan, V., Ingemanson, M.L., Cramer, S.C., Wolbrecht, E.T., Reinkensmeyer, D.J. Robotic Assistance for Training Finger Movement Using a Hebbian Model: A Randomized Controlled Trial. Neurorehabil Neural Repair (2017) 31(8):769–80. doi: 10.1177/1545968317721975.
23. Goodman, R.N., Rietschel, J.C., Roy, A., Jung, B.C., Diaz, J., Macko, R.F., et al. Increased reward in ankle robotics training enhances motor control and cortical efficiency in stroke. J Rehabil Res Dev (2014) 51(2):213–28. doi: 10.1682/JRRD.2013.02.0050.
24. Abe, M., Schambra, H., Wassermann, E.M., Luckenbaugh, D., Schweighofer, N., Cohen, L.G. Reward Improves Long-Term Retention of a Motor Memory through Induction of Offline Memory Gains. Current Biology (2011) 21(7):557–62. doi: 10.1016/j.cub.2011.02.030.
25. Reinkensmeyer, D.J. How to Retrain Movement after Neurologic Injury: A Computational Rationale for Incorporating Robot (or Therapist) Assistance. In: Annual International Conference of the IEEE Engineering in Medicine and Biology – Proceedings (2003). p. 1479–82.
26. Shaphe, A., Shalla, I.H., Al Baradie, R.S., Qasheesh, M. Efficacy of closed loop feedback system with augmented virtual reality visual cues training on gait and functional performance in stroke patients. Biosci Biotechnol Res Commun (2018) 11(1):70–5. doi: 10.21786/bbrc/11.1/10.
27. Vilimovsky, T., Chen, P., Hoidekrova, K., Petioky, J., Harsa, P. Prism adaptation treatment to address spatial neglect in an intensive rehabilitation program: A randomized pilot and feasibility trial. PLoS One (2021) 16. doi: 10.1371/journal.pone.0245425.
28. De Bruyn, N., Saenen, L., Thijs, L., Van Gils, A., Ceulemans, E., Essers, B., et al. Brain connectivity alterations after additional sensorimotor or motor therapy for the upper limb in the early-phase post stroke: A randomized controlled trial. Brain Commun (2021) 3(2). doi: 10.1093/braincomms/fcab074.
29. Doost, M.Y., Herman, B., Denis, A., Sapin, J., Galinski, D., Riga, A., et al. Bimanual motor skill learning and robotic assistance for chronic hemiparetic stroke: A randomized controlled trial. Neural Regen Res (2021) 16(8):1566–73. doi: 10.4103/1673-5374.301030.
30. García-Ramos, B.R., Villarroel, R., González-Mora, J.L., Revert, C., Modroño, C. Neurofunctional correlates of a neurorehabilitation system based on eye movements in chronic stroke impairment levels: A pilot study. Brain and behavior (2023) 13(8), doi: 10.1002/brb3.3049.
31. Gilmore, P.E., Spaulding, S.J. Motor learning and the use of videotape feedback after stroke. Top Stroke Rehabil (2007) 14(5):28–36. doi: 10.1310/tsr1405-28.
32. Hegazy, R.M., Alkhateeb, A.M., Abdelmohsen, A.M. Impact of a virtual reality program on post-stroke upper limb function: a randomized controlled trial. Physiotherapy Quarterly 2022; 30(4):81-86. doi:10.5114/pq.2021.111210
33. Huang, C.Y., Chiang, W.C., Yeh, Y.C., Fan, S.C., Yang, W.H., Kuo, H.C., et al. Effects of virtual reality-based motor control training on inflammation, oxidative stress, neuroplasticity and upper limb motor function in patients with chronic stroke: a randomized controlled trial. BMC Neurol (2022) 22(1). doi: 10.1186/s12883-021-02547-4.
34. Jonsdottir, J., Cattaneo, D., Recalcati, M., Regola, A., Rabuffetti, M., Ferrarin, M., et al. Task-oriented biofeedback to improve gait in individuals with chronic stroke: Motor learning approach. Neurorehabil Neural Repair (2010) 24(5):478–85. doi: 10.1177/1545968309355986.
35. Jonsdottir, J., Cattaneo, D., Regola, A., Crippa, A., Recalcati, M., Rabuffetti, M., et al. Concepts of motor learning applied to a rehabilitation protocol using biofeedback to improve gait in a chronic stroke patient: An A-B system study with multiple gait analyses. Neurorehabil Neural Repair (2007) 21(2):190–4. doi: 10.1177/1545968306290823.
36. Junata, M., Cheng, K.C.C., Man, H.S., Lai, C.W.K., Soo, Y.O.Y., Tong, R.K.Y. Kinect-based rapid movement training to improve balance recovery for stroke fall prevention: a randomized controlled trial. J Neuroeng Rehabil (2021) 18(1):150. doi: 10.1186/s12984-021-00922-3.
37. Kamatchi, K., Jibi, P., Jagatheesan, A., Harikrishnan, N. The Impact of Virtual Reality on Upper Extremity Function In Stroke Patients: A Pilot Study. Journal of Population Therapeutics and Clinical Pharmacology (2023) 30(8), 312–320. doi: 10.47750/jptcp.2023.30.08.033.
38. Kim, H., Kim, J., Jo, S., Lee, K., Kim, J., Song, C. Video augmented mirror therapy for upper extremity rehabilitation after stroke: a randomized controlled trial. Journal of neurology (2023) 270(2), 831–842. doi: 10.1007/s00415-022-11410-6.
39. Kim, G.J., Chen, P. Role of instruction adherence during highly structured robotic arm training on motor outcomes for individuals after chronic stroke. Am J Phys Med Rehabil (2020) 99(4):353–6. doi: 10.1097/PHM.0000000000001333.
40. Krishnamoorthy, V., Hsu, W.L., Kesar, T.M., Benoit, D.L., Banala, S.K., Perumal, R., et al. Gait training after stroke: A pilot study combining a gravity-balanced orthosis, functional lectrical stimulation, and visual feedback. Journal of Neurologic Physical Therapy (2008) 32(4):192–202. doi: 10.1097/NPT.0b013e31818e8fc2.
41. Longatelli, V., Pedrocchi, A., Guanziroli, E., Molteni, F., Gandolla, M. Robotic Exoskeleton Gait Training in Stroke: An Electromyography-Based Evaluation. Front Neurorobot (2021) 15. doi: 10.3389/fnbot.2021.733738.
42. Luque-Moreno, C., Cano-Bravo, F., Kiper, P., Solís-Marcos, I., Moral-Munoz, J.A., Agostini, M., et al. Reinforced Feedback in Virtual Environment for Plantar Flexor Poststroke Spasticity Reduction and Gait Function Improvement (2019). doi: 10.1155/2019/6295263.
43. Maenza, C., Wagstaff, D.A., Varghese, R., Winstein, C., Good, D.C., Sainburg, R.L. Remedial Training of the Less-Impaired Arm in Chronic Stroke Survivors With Moderate to Severe Upper-Extremity Paresis Improves Functional Independence: A Pilot Study. Front Hum Neurosci (2021) 15. doi: 10.3389/fnhum.2021.645714.
44. Maggio, M.G., Naro, A., Manuli, A., Maresca, G., Balletta, T., Latella, D., et al. Effects of Robotic Neurorehabilitation on Body Representation in Individuals with Stroke: A Preliminary Study Focusing on an EEG-Based Approach. Brain Topogr (2021) 34(3):348–62. doi: 10.1007/s10548-021-00825-5.
45. Mazzoleni, S., Tran V.D., Dario, P., Posteraro, F. Effects of Transcranial Direct Current Stimulation (tDCS) Combined With Wrist Robot-Assisted Rehabilitation on Motor Recovery in Subacute Stroke Patients: A Randomized Controlled Trial. IEEE Transactions on Neural Systems and Rehabilitation Engineering (2019) 27(7):1458–66. doi: 10.1109/TNSRE.2019.2920576.
46. Castro-Medina, K.G. Effects of Visual Feedback on Walking Speed for Stroke Patients: Single-case Design. Revista de investigación e innovación en ciencias de la salud (2023) 5(1):127-142. doi: https://doi.org/10.46634/riics.153.
47. Paolucci, T., Agostini, F., Mangone, M., Bernetti, A., Pezzi, L., Liotti, V., et al. Robotic rehabilitation for end-effector device and botulinum toxin in upper limb rehabilitation in chronic post-stroke patients: an integrated rehabilitative approach. Neurol Sci (2021) 42(12):5219-5229. doi: 10.1007/s10072-021-05185-3.
48. Park, M., Ko, M.H., Oh, S.W., Lee, J.Y., Ham, Y., Yi, H., et al. Effects of virtual reality-based planar motion exercises on upper extremity function, range of motion, and health-related quality of life: A multicenter, single-blinded, randomized, controlled pilot study. J Neuroeng Rehabil (2019) 16(1). doi: 10.1186/s12984-019-0595-8.
49. Piron, L., Turolla, A., Agostini, M., Zucconi, C.S., Ventura, L., Tonin, P., et al. Motor learning principles for rehabilitation: A pilot randomized controlled study in poststroke patients. Neurorehabil Neural Repair (2010) 24(6):501–8. doi: 10.1177/1545968310362672.
50. Powers, J., Wallace, A., Mansfield, A., Mochizuki, G., Patterson, K.K. The effect of frequency of feedback on overground temporal gait asymmetry post stroke. Top Stroke Rehabil (2022) 29(6):401–10. doi: 10.1080/10749357.2021.1943796
51. Pundik, S., McCabe, J., Skelly, M., Salameh, A., Naft, J., Chen, Z., et al. Myoelectric Arm Orthosis in Motor Learning-Based Therapy for Chronic Deficits After Stroke and Traumatic Brain Injury. Front Neurol (2022) 13. doi: 10.3389/fneur.2022.791144.
52. Sainburg, R.L., Maenza, C., Winstein, C., Good, D. Motor lateralization provides a foundation for predicting and treating non-paretic arm motor deficits in stroke. In: Advances in Experimental Medicine and Biology. Springer New York LLC (2016). p. 257–72. doi: 10.1007/978-3-319-47313-0_14.
53. Salameh, A., McCabe, J., Skelly, M., Duncan, K.R., Chen, Z., Tatsuoka, C., et al. Stance Phase Gait Training Post Stroke Using Simultaneous Transcranial Direct Current Stimulation and Motor Learning-Based Virtual Reality-Assisted Therapy: Protocol Development and Initial Testing. Brain Sci (2022) 12(6):701. doi: 10.3390/brainsci12060701.
54. Saleh, S., Fluet, G., Qiu, Q., Merians, A., Adamovich, S.V., Tunik, E. Neural Patterns of reorganization after intensive robot-assisted Virtual reality Therapy and repetitive Task Practice in Patients with chronic stroke. Front Neurol (2017) 8. doi: 10.3389/fneur.2017.00452.
55. Schreiber, J., Sober, L., Banta, L., Glassbrenner, L., Haman, J., Mistry, N., et al. Application of motor learning principles with stroke survivors. Occup Ther Health Care (2001) 13(1):23–44. doi: 10.1080/J003v13n01_03.
56. Smedes, F., Giacometti da Silva, L. Motor learning with the PNF-concept, an alternative to constrained induced movement therapy in a patient after a stroke; a case report. J Bodyw Mov Ther (2019) 23(3):622–7. doi: 10.1016/j.jbmt.2018.05.003.
57. Tretriluxana, J., Runnarong, N., Tretriluxana, S., Prayoonwiwat, N., Vachalathiti, R., Winstein, C. Feasibility investigation of the accelerated skill acquisition program (ASAP): Insights into reach-to-grasp coordination of individuals with postacute stroke. Top Stroke Rehabil (2013) 20(2):151–60. doi: 10.1310/tsr2002-151.
58. Tsaih, P.L., Chiu, M.J., Luh, J.J., Yang, Y.R., Lin, J.J., Hu, M.H. Practice variability combined with task-oriented electromyographic biofeedback enhances strength and balance in people with chronic stroke. Behavioural Neurology (2018) 2018. doi: 10.1155/2018/7080218.
59. Turolla, A., Daud Albasini, O.A., Oboe, R., Agostini, M., Tonin, P., Paolucci, S., et al. Haptic-based neurorehabilitation in poststroke patients: A feasibility prospective multicentre trial for robotics hand rehabilitation. Comput Math Methods Med (2013). doi: 10.1155/2013/895492.
60. van Vugt, F.T., Kafczyk, T., Kuhn, W., Rollnik, J.D., Tillmann, B., Altenmüller, E.. The role of auditory feedback in music-supported stroke rehabilitation: A single-blinded randomised controlled intervention. Restor Neurol Neurosci (2016);34(2):297–311. doi: 10.3233/RNN-150588
61. Winstein, C., Kim, B., Kim, S., Martinez, C., Schweighofer, N. Dosage Matters. Stroke (2019) 50(7), 1831–1837. doi: 10.1161/STROKEAHA.118.023603.
62. Zollo, L., Rossini, L., Bravi, M., Magrone, G., Sterzi, S., Guglielmelli, E. Quantitative evaluation of upper-limb motor control in robot-aided rehabilitation. Med Biol Eng Comput (2011) 49(10):1131–44. doi: 10.1007/s11517-011-0808-1.
