# Supplementary material for: A scoping review of scientific concepts concerning motor recovery after stroke as employed in clinical trials
Source: Front Neurol. 2023 Dec 11;14:1221656. doi: 10.3389/fneur.2023.1221656 (PMC10749504; doi:10.3389/fneur.2023.1221656)
Supplement: Supplementary file 3 [file Table_3.docx]

***Supplementary Material – Table 3***

**A Scoping Review of Scientific Concepts Concerning Motor Recovery After Stroke as Employed in Clinical Trials**

**Martina Favetta, Alberto Romano, Nicola Valè, Blazej Cieslik, Sara Federico, Alessia Girolami, Deborah Mazzarotto, Giorgia Pregnolato*, Anna Righetti, Silvia Salvalaggio, Enrico Castelli, Nicola Smania, Stefano Bargellesi, Pawel Kiper and Maurizio Petrarca**

*** Correspondence:** Giorgia Pregnolato: [giorgia.pregnolato@hsancamillo.it](mailto:giorgia.pregnolato@hsancamillo.it)

**Table3.** The characteristics of the studies.

| **Author (year)** | **Study**  **Design** | **Aim** | **Groups characteristics  (n, age, sex)** | **Intervention details** | **Intervention characteristics  (n, frequency, intensity)** | **Outcome**  **measures** | **Findings** |
| --- | --- | --- | --- | --- | --- | --- | --- |
| **Declared** | | | | | | | |
| Dipietro, L., et al. (2009) (1) | Case series | To explore whether untrained UL movements during motor recovery from stroke exhibit changes in smoothness and sub-movements, thereby investigating their nature. | n=47  age (mean)= 57.8y  F/M=16/31 | Training with InMotion ARM robot. | 18 sessions (3 per week/ 6 weeks).  Each session lasted 60min. | - FMA-UE - Circle drawing quality | The shapes drawn by subjects became closer to circles and smoother. Results support sub-movement theory and indicate that smoothness and sub-movements may describe UL motor recovery from stroke, as well as share traits with motor learning. |
| Grimm, F., et al, (2016) (2) | Case series | To assess closed-loop task difficulty adaptation during VR reach-to-grasp UL training assisted with an exoskeleton in stroke rehabilitation. | n=5  age (mean)= 52y  F/M=2/3 | Training with Armeo Spring.  The level of difficulty for the exercise was adjusted by a performance-dependent real-time adaptation algorithm that automated ROM improvement. | 20 sessions | - UL accuracy, temporal efficiency, ROM  -FMA-UE | This approach may facilitate motor learning by progressively challenging the subject in accordance with the individual capacity for UL functional restoration. |
| Grimm, F., et al. (2016) (3) | Case series | To test feasibility and clinical validity of VR visualization and closed-loop feedback of joint-specific movement in severely stroke patients.  To quantify the individual degree of UL natural movement restoration or compensation. | n=3  age (mean)= 62y  F/M=0/3 | Training with Armeo Spring.  The participants tracked and adjusted their movements accordingly with the information provided by the VR environment. They were informed that their movement quality with implicit online feedback. | 20 sessions.  Each session lasted 30 min and consisted of 150 trials. | - FMA-UE  - UL ROM | UL Motor performance (accuracy, temporal efficiency, ROM) and movement quality (proximal inter-joint coordination) improved significantly in stroke patients. |
| Longo, D., et al. (2022) (4) | RCT | To investigate the hypothesis that MSM could influence the modulation of the TSRT.  To investigate the MSM as a rehabilitation technique to improve body functions and activities in individuals with limitations due to chronic stroke. | n=10 | EG: MSM  Dorsal and plantar flexion movements were rhythmically performed for 15 minutes. The participant was requested to relax, avoid any voluntary movement, and let the therapist move the foot.  CG: conventional physical therapy | 4 sessions (1 per week/ 4 weeks)  Each session lasted 40 minutes | Montreal Spasticity Measure Device (EMG and goniometer) | The MSM seems able to modulate the TSRT in individuals with stroke. |
| Petrarca, M., et al. (2011) (5) | Case report | To move in the direction of a person-oriented decision-making process on gait rehabilitation by analyzing the recovery from stroke of a six-year-old boy over three years. | n=1  age= 6 y | Phase T1: Imposition of traction stress therapeutic maneuvers during passive muscle shortening with an ad hoc platform.  Phase T2: cognitive and selective motor joint control.  Phase TT: stretching, muscle reinforcement and walk indoors on even and level surfaces.  Phase T3 and TT: generic treatment and training selective body segments during the interaction with the environment while the subject executed a task oriented towards a functional goal. | 3 years of training:  phase T1: exercises performed 3 times, for 20 minutes, over six days.  Phase T2: 1 hours a day, six days a week.  Phase TT:1 hour per day, 3 times per week.  Phase T3: 4 months  Phase TT: 12 months  Phase T3: 12 months | -kinetics and kinematics (Gait Analysis)  -GMFM  -PEDI | Treatment T1 seems to be correlated with an improvement of muscle recruitment; treatment T2 is associated with gait recovery; and finally, treatment T3 is linked with functional pattern change. |
| Pollock, C.L., et al. (2014) (6) | Case series | To retrain of multidirectional stepping reactions may be informed by the CPF to improve balance function in people with stroke. | n= 4  age [min-MAX]= [53– 68] y | Protective Stepping Reaction Retraining Protocol: the patients had to past the perceived limits of stability as a stimulus to produce a stepping reaction in multiple directions to prevent a fall. Participants completed 2 sets of 60 stepping reactions (10 repetitions with each leg in each direction)  Progression of functional task difﬁculty. Augmented feedback provided to adjust the optimal challenge. | 12 sessions (3 per weeh/ 4 weeks)  Each session lasted 45 min. | - Walking balance Community Balance and Mobility Scale  - ABC scale  - Kinematics (Gait analysis) | Participants improved community-level walking balance. Aspects of balance-related self-efﬁcacy and movement. Kinematics also showed improvements. |
| Reinkensmeyer, D.J., et al. (2009) (7) | RCT | To demonstrate that different dose-matched, UL rehabilitation training techniques can result in similar improvement in movement ability after stroke. | EG  n= 13  CG:  n= 14 | EG: robotic training  CG: unassisted movement training | 24 sessions (3 per week/ 8 weeks)  Each session lasted 60 min. | - strength (shoulder flexion/elbow extension)  - speed  - coordination | Training with or without robotic assistance produces a similar time-course and magnitude of UL motor recovery after chronic stroke. |
| Rowe, J.B., et al. (2017) (8) | Case series | To determine the therapeutic effects of high and low levels of robotic assistance during finger training | Group1  n = 15  age (mean)= 56y  F/M= 6/9;  Group2  n = 15  age (mean)= 60y  F/M= 4/11 | Patients played Guitar Hero with FINGER robotic exoskeleton.  The training protocol varied the amount of robotic assistance while controlling the number, amplitude, and exerted effort of fingers movements.  Participants were randomized to receive high assistance (82% success) or low assistance (55% success). | 9 sessions (3 per week/ 3 weeks)  Patients played 5 songs 2 times each. | - BBT  - NHPT  - NIHSS  - FMA-UE  - ARAT  - Lateral Pinch Strength Test  - Motivation  - self efficacy | Both groups improved significantly on functional and impairment-based motor outcomes, on depression scores, and on self-efficacy of hand function.  Individuals with impaired finger proprioception at baseline benefited less from the training.  High assistance boosted motivation, as well as secondary motor outcomes (FMA-UE and Lateral Pinch Strength) – particularly for individuals with more severe finger motor deficits. |
| Shaphe, A., et al. (2018) (9) | RCT | To investigate the efficacy closed loop visual cues incorporated augmented VR environment on functional gait and community ambulation in stroke patients. | EG  n= 14  age (mean)= 42.7y  CG:  n= 14  age (mean)= 46.5y | EG: physical therapy session + Closed loop visual cue augmented VR training  CG: physical therapy session + traditional gait training | 24 sessions (6 per week/4 weeks) | - Gait Analysis  - SF-SIS | The finding supported the beneficial effect of augmented VR based closed loop visual cue training for improving the gait and functional ambulation in stroke patients. The improvement may be attributed to the determined efforts of the patients in response to the closed loop visual cues for effectively controlling their lower limbs. |
| Vilimovsky, T., et al. (2021) (10) | RCT | PAT reduced visuospatial symptoms of spatial neglect among patients in an inpatient setting providing intensive rehabilitation care.  PAT enhanced the recovery of spatial neglect. | EG  n= 12  age (mean)= 51.5y  F/M=7/5  CG  n= 11  age (mean): 58  F/M= 6/5 | EG: PAT treatment: patient wore goggles fitted with 20-diopter prism lenses that shift the visual field to the ipsilesional side of space for 11.4 degrees of visual angle.  CG: Sham treatment: used flat goggles that did not shift the visual field at all.  During each session, participants completed 60 visuomotor movements while the first part of arm movements was blocked from view. | 10-session (5 per week/2 weeks).  Each session lasted 15–20 min. | - CBS  - Bells test  - Line bisection  - Scene copying test | No evidence that PAT specifically reduced spatial neglect symptoms or enhanced spatial neglect recovery.  Both PAT and Sham treatment may improve visuospatial ability and function among individuals with spatial neglect after unilateral brain damage.  PAT not particularly effective in ameliorating spatial neglect in the clinical setting that offers highly intensive rehabilitation program. |
| **Not Declared** | | | | | | | |
| De Bruyn, N., et al. (2021) (11) | RCT | To investigate differences in therapy-induced resting-state functional connectivity changes between additional sensorimotor therapy compared with motor therapy in the early-phase post stroke | EG: n= 18  CG: n= 12 | EG: rehabilitation care + sensorimotor therapy CG: rehabilitation care + motor therapy | 16 sessions (4 per week/4 weeks).  Each session lasted 60min. | - fMRI | The sensorimotor and motor therapy group showed no significant differences in terms of pre-to-post changes in inter-hemispheric connectivity or ipsilesional intrahemispheric connectivity. |
| Doost, M.Y., et al. (2021) (12) | RCT | To investigate whether training under the robotic active-assisted mode improves bimanual motor skill learning more than training under the active mode in stroke patients. | EG: n= 18  CG: n= 12 | Robotic training (REAplan®)  EG: active mode CG: active-assisted mode | 2 sessions.  Intervention lasted 2 days. | - Speed  -accuracy | The stroke patients generalized the learned skill only in the active subgroup. |
| García-Ramos, B. R., et al. (2023) (13) | Case series  Pilot study | To explore the effect of a new game-based ocular virtual reality training on the cerebral activity in sensorimotor  regions and accuracy in eye and hand movements in three different profiles of stroke survivors. | n=3  age (mean)= 67.3y  F/M=1/2 | Eye movements accuracy training through eye-tracker controlled games (memory, whac-a-mole, adventure game and target shooting). | 28 daily sessions (7 per week for 4 weeks) at the participants houses. Each session lasted 20-30 minutes. | - FMA-UE;  - mean absolute error during the continuous tracking of a target task mentioned above;  - fMRI scanning during continuous tracking of a target tasks through eye-tranking and a hand-controlled joystick. | No change above the minimum clinically important difference occurred in the FMA-UE. The mean absolute error decreased in the eye- and hand-controlled fMRI task in two of the three participants.  During the fMRI task, the contrasts in the eye-controlled and in the hand-controlled runs show neural activity increases after the training period, particularly in the premotor cortex, primary motor area, cerebellum, basal ganglia, putamen, and insula.  Training on the ocular control of  virtual objects can be a useful tool to increase neural activity in motor  areas of interest that may be useful for neurorehabilitation in clinical  practice and generate mechanisms to promote motor learning or  motor transfer. |
| Gilmore, P.E., & Spaulding, S.J. (2007) (14) | RCT | To determine the effectiveness of combining a program of VOT and OT in learning the skill of donning socks and shoes poststroke | EG:  n= 5  age (mean)= 65.8y  CG:  n= 5  age (mean)=72y | EG: VOT  CG: OT | 10 maximum sessions.  Treatment was considered successful and was stopped if the participant was able to independently do his or her socks and shoes prior to the 10 session maximum. | -KB-ADL socks and shoes subtests.  - COPM | This study found no significant differences between the OT and VOT groups in motor learning. Motor learning had occurred for both groups regardless of the feedback method used. The VOT group participants indicated that they felt they performed better and they were more satisfied with their performance than the OT group. |
| Hegazy E. M. et al. (2022) (15) | RCT | To compare the effect of a virtual reality training program and a task-oriented training program on the paretic upper limb function after stroke. | EG:  n=10  age (mean)= 54.2y  CG:  n=10  age (mean)=56.4y | EG: VR + task-oriented training program  CG: task-oriented training program | 18 sessions (3 per week).  Each session lasted 60 min. In EG, the VR training activities lasted 15 minutes. | -UEFI  -grip strength test (using a dynamometer) | Significant improvements in the EG post-intervention (both in UEFI and grip strength) compared with CG (p < 0.05). |
| Huang, C.Y., et al. (2022) (16) | RCT | To identify the effects of immersive VR training on inflammation, oxidative stress, neuroplasticity and UL motor function in stroke patients. | EG:  n= 15;  age(mean)= 50.80y  F/M= 9/6  CG:  n= 15  age(mean)= 58.33y  F/M= 11/4 | EG: VR training VRbased activities. Six to ten tasks were assigned in each session  CG: conventional OT | 16 sessions (2-3 per week).  Each session lasted 60 min. | -FMA-UE  -AROM (shoulder flexion, elbow extension, wrist extension, forearm supination and pronation)  -SSQ  -RPE molecular biomarkers (BDNF) | Significant improvements in overall UL function and AROM were found after the intervention, while only elbow extension and forearm pronation were significantly different between groups (higher improvement for VR group).  Moreover, significant improvement of BDNF expression level was found after intervention in the VRT group. |
| Jonsdottir, J., et al. (2010) (17) | Case report | To examine the efficiency of EMG BFB training combined with theories of motor learning in improving performance and learning of gait parameters after stroke. | n= 1  age(mean)= 55y  F/M= =71 | EMG BFB was applied to the gastrocnemius lateralis. An auditory feedback tone was used to indicate whether push-off power met the target. | 20 sessions (3 per week).  Each session lasted 45 min. | -Gait analysis | Continuous feedback and blocking, that is, constant feedback applied only during normal walking in the beginning, may have enhanced fundamental spatial-temporal patterning. |
| Jonsdottir, J., et a.l. (2007) (18) | RCT | To evaluate the efficacy of task-oriented EMG-BFB in increasing push off power of the plantar flexors muscle on the affected side and to increase gait velocity in a population with hemiparetic stroke. | EG:  n= 10  age(mean)= 61.6y  CG:  n= 10  age(mean): 62.6y | EG: EMG BFB consisted of an acoustic signal driven by the EMG recorded from the gastrocnemius lateralis during gait.  CG: usual rehabilitation care | 20 sessions (3 per week).  Each session lasted 45 min. | -Ankle power peak (W/kg)  -Velocity  - Stride length  -Knee flexion peak (degrees) | The EG increased peak ankle power and, concurrently, gait velocity and stride length in a population with chronic hemiparesis, suggesting that intervening at the impairment level is effective in promoting functional improvement. |
| Junata, M., et al. (2021) (19) | RCT | To evaluate the effectiveness of the interactive RMT and CBT on chronic stroke survivors overall balance recovery reaction. | EG:  n=16  age (mean)=60.6y  F/M=12/4  GC:  n=14  age (mean) 60.1y  F/M=2/12 | EG: RMT requires patients to do rapid movement to quickly reach out a target in different directions by hand, or make a quick step to restore balance during a fall incidence.  CG (2): CBT | 20 sessions (3 per week/ for 7 weeks)  Each session lasted 60 min. | -BBS  -TUG  -FMA-UE  -Barthel index  -EMG activation | RMT results in improved overall balance in chronic stroke patients by improving automatic postural responses and reactive stepping performance. |
| Kamatchi K. et al. (2023) (20) | Longitudinal pilot study | To assess the effectiveness of virtual reality in the rehabilitation of persons with stroke to improve the upper extremity function. | n=10  age (range)=45-70y  F/M=n.a. | Training with immersive virtual reality with a headset and a manual controller (I.e., IRSU PLAY VR) | 24 sessions (3 per week/8 weeks).  Each session lasted 45 minutes. | - FMA-UE  - sEMG signal amplitude | The FMA-UE score improved significantly after the intervention.  Signal amplitude recorded with sEMG significantly improved. |
| Kim H., et al. (2023) (21) | RCT | To investigate the effects of mirror therapy with video augmented wearable refection device on reach-to-grasp motor control and upper extremity motor function. | EG:  n=12  age (mean)=59.6y  F/M=5/7  CG1:  n=12  age (mean) 61.0y  F/M=4/8  CG2:  n=12  age (mean) 58.8y  F/M=5/7 | EG: video augmented wearable refection device  CG1: traditional mirror therapy  CG2: conventional rehabilitation | 20 sessions (5 per week/ for 4 weeks). Each session lasted 30 min. | -Trunk kinematics  -FMA-UE  -MFT  -BBT | The results suggested that mirror therapy using a video augmented wearable refection device is more efficient compared to traditional mirror therapy for patients with stroke. |
| Kim, G.J., & Chen, P. (2020) (22) | Case series | To examine the effects of instruction adherence on UL motor outcomes after highly structured intervention. | n=30  age (mean)58.1 y  F/M=16/14 | Training with InMotion ARM robot. | 12 sessions (4 weeks). | -MCQ -FMA-UE -WMFT  -FAS | Instruction adherence did not affect motor improvement. Adherence to explicit information may play a limited role in motor learning for stroke survivors with moderate-to-severe arm impairment during highly structured training protocols. |
| Krishnamoorthy, V., et al. (2008) (23) | Case report | To describe the findings of training with a specially designed gravity-balanced orthosis, treadmill walking, FES, and principles of motor learning. | n=1  age (mean)= 58y  F/M=0/1 | Gait retraining during which the gravity compensation provided by the gravity-balanced orthosis and visual feedback about walking performance was gradually reduced. | 15 sessions (5 per week/ 3 weeks). Each session lasted 40 min. | -TUG  -FMA-LE  -BBS  -Gait analysis | This approach showed positive effects on the participant’s gait. Advantages include precise computer-controlled training parameters and feedback, and accurate data to monitor progress across sessions. |
| Longatelli, V., et al. (2021) (24) | Single-blinded pilot study | To investigate the gait rehabilitation process and evaluates motor re-learning in patients with subacute post-stroke by analysing and comparing LL muscular activation patterns. | EG:  n= 15  age(mean)=65y  F/M=5/10  CG:  n= 14  age (mean)= 68y  F/M=3/11 | EG: conventional therapy + exoskeleton-assisted gait  CG: conventional therapy + conventional gait training | 20 sessions (3 per week/ 4 weeks). For Each session lasted 60 min. | - Gait functionality (by means of clinical scales combined to obtain a Capacity Score (5-item modified Barthel index, MI, TMWT, 6MWT, FAC, TCT).  - Gait neuromuscular lower limbs pattern using surface EMG signals | Both groups improved their ability to walk in terms of functional gait. However, only the EG regained a rhythmic and controlled gait, as observed by the muscular activation patterns of proximal LL muscles, inducing the patient to regain a more physiological gait. |
| Luque-Moreno, C., et al. (2019) (25) | Case series | To determine whether a program that includes the combination of TR and RFVE decreases the level of spasticity of the PF muscles and improved gait function | n=10  F/M=0/10 | EG: stand on the non-paretic limb and perform open kinetic trajectories with the paretic limb in VR scenario  CG: TR of LL (passive, assisted, and active exercises). | 15 sessions (5 per week/ 3 weeks). For Each session lasted 60 min. | - MAS  - FAC  - FIM | The mean FAC scores increased indexing an improvement in ambulation functionality.  The mean MAS scores decreased thus indicating reduced spasticity treatment. FIM score increased scale were observed, although it did not reach the significance level. |
| Maenza, C., et al. (2021) (26) | Longitudinal pilot study | To assess whether a rehabilitation approach focused on remediation of ipsilesional arm motor deficits in stroke survivors with moderate to severe contralesional arm paresis and with significant ipsilesional arm coordination deficits, improves functional performance and independence. | n=13 | Period 1: Ipsilesional Arm Therapy Sessions: VR games Kinereach for target reaching,  Period 2: Sham Sessions:  real life dexterity training with resistive warm up theraband and then a series of 6 real life tasks. | Both for period 1 and 2, 9 sessions were provided (3 per week/3 weeks).  Each session lasted 90 min. | - JTHFT  - FIM  - Grip Strength hand dynamometer  - FMA-UE | Ipsilesional arm training significantly improves ipsilesional arm motor performance (JTHFT) and generalizes to improve functional independence (FIM). Participants maintained the improvements 3- and 6-weeks post ipsilesional arm training. Finally, the contralesional arm showed small, yet significant improvements on an impairment measure following ipsilesional arm training.  The training would not be detrimental to the contralesional arm (FMA-UE).  No significant improvements in grip strength. |
| Maggio, M.G., et al. (2021) (27) | RCT | To evaluate the usefulness of a RAGT equipped with augmented visuomotor feedback in improving LL sensorimotor function, gait performance and body representation.  To understand the putative neurophysiological correlates (EEG analysis) of the correlation between BR recovery and motor performance improvement. | EG:  n= 30  age(mean)= 50.4y  F/M= 11/19  CG:  n= 15  age (mean)= 48.2y  F/M= 8/7 | EG: Lokomat®Pro (RAGT + VR) equipped with augmented visuomotor feedback  CG: Lokomat®Nanos (RAGT − VR) not equipped with augmented feedback | 40 sessions (5 per week/ 8 week).  Each session lasted 60 min. | - BES  - BUT  - FMA-LE  - FAB  - MoCA  - BDI  - SF12  - EEG | Results confirms the usefulness to use augmented visuomotor feedback in robotic rehabilitation for improving gait performance in patients with neurological disorders.  Motor and BR improvement were significantly correlated, as suggested by the clinical and EEG data in the patients provided with the visual feedback of a human avatar while walking (RAGT + VR) compared to the control group (RAGT − VR).  The MNS may play a relevant role in BR recovery and motor performance improvement in stroke patients through the observation of gait using augmented visuomotor feedback, resembling VR. |
| Mazzoleni, S., et al. (2019) (28) | RCT | To investigate the effectiveness of combining tDCS and wrist robot-assisted rehabilitation in subacute stroke patients in comparison with the wrist robotic training only | EG:  n= 20  age (mean)= 67.5y  F/M= 12/8  CG:  n= 19  age (mean)= 68.74y  F/M= 12/7 | EG: robot-assisted wrist rehabilitation + tDCS.  The robot-assisted rehabilitation consists of wrist training based on goal-directed reaching tasks by using the InMotion WRIST robot. TDCs: anodal electrode in M1 of the affected hemisphere, cathodal in M1 in other hemisphere, stimulation during the first 20 minutes of training.  CG: robot-assisted rehabilitation + sham tDCS | 30 sessions (5 per week/ 6 week).  Each session lasted 30 min. | - FMA-UE  - MAS (wrist muscles)  - MI  - BBT  - Kinematic parameters of wrist movements: abduction, adduction, extension, flexion | No significant differences between two groups were found. There is the possibility that the relative timing of delivering the stimulation and the robotic therapy may influent the effectiveness of the robotic training. |
| Castro-Medina K.G. (2023) (29) | Single-Case | To determine the effect of visual feedback on gait speed after stroke in adults with subacute and chronic stages. | n=4  Age (mean)=50y  F/M=3/1 | Training with Lokomat.  The participants receive 12 to 20 sessions with robotic gait training without feedback (control) before starting robotic training with visual feedback (intervention). | 25 sessions (3 per week).  Each session lasted 60min. | - TMWT | The robotic training with visual feedback improved gait performance in temporal terms, specifically gait speed. |
| Paolucci, T., et al. (2021) (30) | Prospective observational case-control study | To determine the effects of an integrated rehabilitation protocol, including botulinum toxin and conventional rehabilitation exercise plus end-effector robotic training for UL functional recovery compared to training with the robot alone in post-chronic stroke | EG:  n= 23  age (mean)= 65.7y  F/M= 13/10  CG:  n= 21  age (mean)= 65y  F/M= 7/14 | EG: robotic treatment with end-effector + conventional treatment  CG: robotic treatment + conventional treatment + botulinum toxin for UL recovery. | 20 sessions (3 per week).  Each session lasted 30 min.  Robotic therapy was started seven days after botulinum toxin infiltration | - FMA-UE  - MI  - MAS  - Numeric rating scale. - BBT  - FAT  - Barthel Index | Our results suggested a good efficacy in the reduction of spasticity and in the UL function improvement, with the reduction of UL pain, adopting a rehabilitation protocol integrated with botulinum toxin infiltration, robot-assisted training, and traditional physiotherapy. |
| Park, M., et al. (2019) (31) | RCT | To assess the clinical effectiveness of the VR-based rehabilitation device (Rapael Smart Board™) for the UL rehabilitation in chronic stroke patients.  To investigate the correlations between kinematic data from the Rapael Smart Board™ and clinical outcome. | EG:  n= 12  age (mean)=53.5y  F/M: 5/7  CG:  n= 13  age(mean)= 51.5y  F/M: 5/8 | EG: Rapael Smart Board™  CG: conventional treatment | 20 sessions (5 per week/ 4 weeks).  Each session lasted 60 min. | - FMA-UE  - WMFT  - AROM (proximal upper extremities)  - Barthel index  - SIS | All functional outcome measures (FMA, WMFT, and Barthel Index) showed significant improvements in the EG and CG. The EG was available for improving UL function and health-related quality of life and useful for assessing UL ability in stroke survivors. |
| Piron, L., et al. (2010) (32) | Single blind RCT | To determine if a rehabilitation technique that aimed to augment the possibility of motor learning using VR (RFVE) could improve motor outcome scores significantly more than conventional treatment.  To compare the effects of a VR based technique with a control intervention of progressive therapy for the affected UL. | EG:  n= 27  age (mean)= 58.8y  M/F= 17/10  CG:  n= 23  age (mean)= 62.2y  M/F 12/11 | EG: perform different kinds of motor tasks while the movement of the entire bio-mechanical arm system's end section (end effector) was simultaneously represented in a virtual scenario by means of motion-tracking equipment.  CG: specific exercises with the UL with progressive complexity based on Bobath principles. | 20 sessions (5 per week/ 4 weeks).  Each session lasted 60 min. | - FMA-UE  - ROM (Shoulder, Elbow, Wrist)  - AROM  - PROM  - MAS  - CAHAI  - CHART  - EMG analysis | Both rehabilitation therapies improved arm motor performance and functional activity, but the RFVE therapy induced more robust results in patients exposed to late rehabilitation treatment. |
| Powers, J., et al. (2022) (33) | RCT | To investigate the effects of augmented feedback during overground gait training, on TGA | Group1:  n=8  age [min-MAX]= [44-81]y  F/M=4/4  Group2:  n=6  age [min-MAX]= [56-83]y  F/M=2/4  Group3:  n=4  age [min-MAX]= [56-75]y  F/M=0/4 | Group1: 100% feedback  (after every walking trial)  Group2: ~50% feedback  (after every second trial)  Group3: 0% feedback  (no feedback given) | 2 sessions.  Each session lasted time to do 25 tasks. | - NIHSS  - MCA  - TGA  - gait speed | Visual feedback delivered at a high frequency (Group1) during a single session of overground walking can change TGA post-stroke without reducing gait speed. |
| Pundik, S., et al. (2022) (34) | Pilot mixed cohort interventional study | To evaluate MyoPro as a tool for motor learning-based therapy for individuals with chronic UL impairment. | n=7  age(mean)= 65.4y  F/M= 5/2 | Treatment consisted of a combination of MyoPro training ML therapy. Tasks included grasp/release, hand to mouth movements, forward reaching movements, bimanual tasks, and fine motor manipulation of objects. | 18 sessions (2 per week/9 weeks)  + home exercise program (9 weeks). | - FMA-UE; Shoulder, Elbow, Wrist  - PROM/AROM  - MAS  - CAHAI  - CHART  - OPUSsat | Use of MyoPro in ML resulted in clinically significant gains with a relatively short duration of in-person treatment. |
| Sainburg, R.L., et al. (2016) (35) | Case series | To test intense non-paretic arm training in improving motor coordination and functional performance in the trained arm, but also in improving functional independence and paretic arm function. | n=3  F/M=0/3  Age (mean)= 66.3y | VR based movement training with feedback displayed on a horizontal mirror positioned above the table surface. This mirror reflected the stimuli presented on a horizontal, inverted. The proximal interphangeal joint of the index finger was reflected by the position of the cursor.  Six Degree of freedom Trackstar® magnetic sensors were attached to the limbs. | 9 sessions (3 per week, for 3 weeks). Each session lasted 90min. | - FIM  - FMA-UE  - JTHFT | Substantial improvements in ipsilesional arm movement kinematics, functional performance, and that these improvements carried over to improve functional independence. |
| Salameh, A., et al. (2022) (36) | Pilot - preliminary study | To develop and test a combination protocol of simultaneous brain stimulation and focused stance phase training for people with chronic stroke. | n=5  age(mean)= 58.6y  F/M=0/5 | Gait recovery via tDCS, Treadmill and VR during specific functional treatment. | 10 sessions (5 per week/2 weeks)  Each session lasted 60 min. | - TMWT  - TUG  - Functional Gait Assessment | It was feasible to administer tDCS simultaneously with highly focused VR treadmill gait training for individuals with chronic stance phase deficits after stroke. |
| Saleh, S., et al. (2017) (37) | non-RCT | To compare the effect of robot-assisted VR repetitive task practice-based interventions on neural pattern reorganization. | EG:  n=9  age (mean)= 57y  F/M= 3/6  CG:  n=10  age (mean)= 59.6y  F/M= 8/2 | EG: robot-assisted VR training. Reaching tasks to virtual targets and objects in 3D space.  CG: repetitive task practice with robotic-assisted training, without VR | 8 sessions (4 per week/ 2 weeks).  Each session lasted 180 min. | - fMRI  - JTHFT | Each group showed distinct patterns of reorganization. The EG exhibited better improvement on clinical measures. Brain reorganization in the CG suggested an adaptive compensatory process in the contralesional hemisphere, while improvement in the EG was attributed to reinstatement of activity in ipsilesional sensorimotor networks. |
| Schreiber, J., et al. (2001) (38) | Case series | To determine the relationship between type of task and type of environment on retention and transfer of motor skills when applied to stroke survivors, as measured by time to complete the task and the number of errors. | n= 4 | Self-created keyboard typing task aimed at motor learning including the acquisition and retention trials, and proprioceptive feedback. | No clear information. | - Number of errors in keyboard typing. | There was no consistent relationship found between the percent change in time and errors data. Therefore, trends regarding the subjects’ typing performance during the motor learning phases could not be identified. |
| Smedes F., & da Silva, L.G. (2019) (39) | Case report | To illustrate the clinical reasoning and the feasibility of applying PNF-concept as an alternative approach in patients who are not accepted or not suitable for CIMT. | n=1  age (mean)= 68 y | Task-oriented motor re-learning exercises with the specific use of PNF-patterns focused on reaching and grabbing activities used in daily life. Verbal, visual and tactile stimulus, resistance, approximation, traction, stabilizing, stretch and relax were within PNF patterns. | 6 weeks. | - AROM/PROM wrist extension  - grip strength (handheld dynamometer)  - MAS  - FAT  - NHPT | PNF movement patterns provided beneficial effects in cortical adaptations and cortical organization resulting in motor learning effects. In cases CIMT is difficult to apply, a specified PNF-based therapy has shown to be a feasible alternative. |
| Tretriluxana, J., et al. (2013) (40) | Case series | To investigate the feasibility of Accelerated Skill Acquisition Program delivered during the 1- to 3-month outpatient interval in stroke survivors and included an assessment of Reach-to-Grasp coordination. | n= 6 | Individualized arm therapy program based on the Accelerated Skill Acquisition Program. | 15 session (2-3 per week/5-8 weeks). Each session lasted 120 min. | - Reach-to-Grasp  - WMFT  - SIS | Under Accelerated Skill Acquisition Program, WMFT tasks and Reach-to- Grasp actions were performed faster with higher peak transport velocity and a more coordinated Reach-to-Grasp pattern. |
| Tsaih, P.L., et al. (2018) (41) | RCT | To determine the effects of constant force or variable force practice with task related EMGBFB-assisted exercise training on the TA muscle strength, balance, and LL motor function in people with chronic stroke. | EG1:  n=11  age [min-MAX]= [26-68]y  F/M=1/10  EG2:  n=13  age [min-MAX]= [29-74]y  F/M=4/9  CG:  n=9  age [min-MAX]= [42-66]y  F/M=2/7 | EG1: general PT + variable practice EMG BFB (contract the TA muscle to match the maximal EMG signal)  EG2: general PT + constant practice EMG BFB + (var the force outputs of the TA muscle to match EMG BFB signals)  CG: UL exercise without EMG BFB + general PT  Each session was divided into component I (static seated position) and component II (during walking and balance-related activities). | 18 sessions (6 per week/3 weeks).  Each session lasted 40 min. | - TA strength  - dynamic posturography  - walking speed  - TUG  - 6MWT | Task-oriented EMGBFB-assisted TA exercise training improved muscle strength in people with chronic stroke.  EG facilitated improvements in the ability to sway in the anteroposterior direction while standing. |
| Turolla, A., et al. (2013) (42) | A feasibility prospective multicentre trial (uncontrolled) | To evaluate the efficacy of the proposed technological solution for the rehabilitation of hand and fingers motor function in poststroke patients | n= 15  age (mean)= 54y  F/M= 8/7 | Patients were asked to move a finger holder in the 3D space avoiding objects showed in the VR environment. | 15 sessions (5 per week/3 weeks).  Each session lasted 45 min. | - FMA-UE  - NHPT  - speed  - smoothness (Jerk)  - fMRI | FMA-UE and NHPT increased significantly, kinematic parameters improved, post-intervention re-lateralization of cortical activation during exercises. |
| Van Vugt, F.T., et al. (2016) (43) | RCT | To test the hypothesis whether rehabilitation benefits in music-supported therapy are due to auditory feedback-based motor learning. | EG: Jitter group  n= 19  CG: normal group  n=15 | EG: music-supported therapy on a piano that emits sounds after a jittered delay.  CG: music-supported therapy on a piano that emits sounds immediately | 10 sessions.  Each session lasted 30 min. | - Barthel index  - NHPT  - Finger tapping measurements  - Auditory and auditory-motor tests | Auditory feedback-based motor learning is not the beneficial mechanism of music-supported therapy. Immediate auditory feedback therapy may be suboptimal. Jittered delay may increase efficacy of the proposed therapy and allow patients to fully benefit from motivational factors of music training. |
| Winstein, C., et al. (2019) (44) | RCT | To explore the dose response and test the dosage of task-specific practice needed to achieve meaningful  improvement in arm and hand use in chronic stroke  survivors. | Group1: n=10  Group2: n=10  Group3: n=10  Group4: n=11 | The Accelerated Skill Acquisition Program (ASAP) is a personalized task-oriented training (skill acquisition, capacity building). Each module consists of practice pursuant of skill, progression in difficulty and self-assessment with problem solving barriers to improvement. | Therapy was provided in 3 weeklong bouts of 4 consecutive visits each separated by 1 month.  4 dosages of arm and hand practice:  Group1: 0 min dose  Group2: 15 min dose  Group3: 30 min dose  Group4: 60 hour dose | - MAL  - WMFT | Our primary findings demonstrate that the higher the dosage of ASAP, the greater the change in MAL over 3 spaced training bouts (≈4 months) in chronic stroke survivors. Dosage modified the participation-level outcome MAL,  a measure of arm and hand use, but not the activity-level outcome  WMFT, a measure of functional capacity. |
| Zollo, L., et al. (2011) (45) | Case series | To provide quantitative measure of biomechanical and motion planning features of arm motor control following upper-limb robot-aided motor therapy. | n=24  age(main)=55.9y | Robot-aided motor therapy. Point-to-point movements from the center to eight outbound targets. The assistance was tuned based on patient’s performance. In wrist training, the first two assisted games trained wrist flexion/extension, abduction/adduction, and combination of these movements. The last assisted game exercised exclusively pronation and supination. | 36 sessions (3 per week/12 weeks - 6 weeks of shoulder–elbow therapy and 6 weeks of wrist therapy).  Each session lasted 3 times/week. | - FMA-UE  - Motor Power | Robot-aided motor therapy led to significant reduction in motor impairment of the paretic limb from admission to discharge. Patients globally improved kinematic and dynamic performance and all indices varied in the expected direction with statistically significant changes. As regards dynamic performance, the amount of total force and total work directed towards the target (namely the useful force and the useful work) significantly increased in unperturbed as well as in perturbed motion, as a consequence of the combined improvement of motion direction and force regulation. |

**Abbreviations list**: n=number; UL= Upper Limb; y= years old; M= Male; F= Female; y= years old; FMA-UE= Fugl-Meyer Assessment–Upper Extremity; VR= Virtual Reality; ROM= range of motion; RCT= Randomized Controlled Trial; MSM= Muscle Shortening Maneuver; TSRT=Tonic Stretch Reflex Threshold; EG= Experimental Group; CG= Control Group; EMG= electromyography; GMFM= Gross Motor Function Measure; PEDI= Pediatric Evaluation of Disability Inventory; CPF= Challenge Point Framework; ; ABC scale=Activities-speciﬁc Balance Conﬁdence Scale; BBT= Box and Blocks Test; NHPT= Nine Hole Pegboard Test; NIHSS= NIH Stroke Scale; ARAT= Action research arm test; SF-SIS= stroke impact scale; PAT= Prisma Adaptation Treatment; CBS= Catherine Bergego Scale; fMRI= functional Magnetic Resonance Imaging; VOT= videotape feedback occupational therapy; OT=occupational therapy; KB-ADL=Klein Bell Activities of Daily Living Scale; COPM=Canadian Occupational Performance Measure; UEFI= Upper Extremity Functional Index test; AROM= range of active motion; SSQ= Simulator Sickness Questionnaire; RPE= Borg Scale of Perceived Exertion; BDNF= Brain-derived neurotrophic factor; BFB= biofeedback; RMT= Rapid Movement Training; CBT= Conventional Balance Training; BBS= Berg Balance Scale; TUG= Timed Up-and-Go test; MCQ= Manipulation Check Questionnaire; WMFT= Wolf Motor Function Test; FAS=Functional Ability Scale; FES= Functional Electrical Stimulation; FMA-LE= Fugl-Meyer Assessment–Lower Extremity; LL= Lower Limb; MI= Motricity Index; TMWT= ten meters walking test; 6MWT= six minutes walking test; FAC= Functional Ambulatory Category; TCT= Trunk Control Test; TR= traditional rehabilitation; RFVE= reinforced feedback virtual environment; PF= plantar flex; MAS= Modified Ashworth Scale; FAC= Functional Ambulatory Category; FIM= Functional Independence Measure; JTHFT= Jebsen Taylor Hand Function Test; RAGT= robot-aided gait training; EEG= electroencephalogram; BR= body representation; MNS= Mirror Neuron System; BES=Body Esteem Scale; BUT= Body Uneasiness Test; FAB= frontal assessment battery; MoCA= Montreal Cognitive Assessment; BDI= Beck depression inventory; SF12= Short Form-12 health status questionnaire; FAT= Frenchay arm test; tDCS= Transcranial direct current stimulation; MI= Motricity Index; SIS= Stroke Impact Scale; PROM= Passive ROM; CAHAI= Chedoke Arm and Hand Activity Inventory; CHART= Craig Handicap Assessment and Rehabilitation Technique; MCA= Chedoke McMaster Stroke Assessment; TGA= Temporal Gait Asymmetry; ML=motor-learning base therapy; OPUSsat= Orthotic and Prosthetic User’s Survey Satisfaction; TMWT= ten meters walking test; PNF= proprioceptive Neuromuscular Facilitation; CIMT= Constraint Induced Movement Therapy; TA= Tibial Anterior; 6MWT= six minutes walking test; MAL= Motor Activity Log; BART= Bilateral Arm Reaching test.

REFERENCES

1. Dipietro, L., Krebs, H.I., Fasoli, S.E., Volpe, B.T., Hogan, N. Submovement changes characterize generalization of motor recovery after stroke. Cortex. (2009) 45:318–24. doi: 10.1016/j.cortex.2008.02.008
2. Grimm, F., Naros, G., Gharabaghi, A. Closed-Loop Task Difficulty Adaptation during Virtual Reality Reach-to-Grasp Training Assisted with an Exoskeleton for Stroke Rehabilitation. Front Neurosci (2016) 15;10. doi: 10.3389/fnins.2016.00518.
3. Grimm, F., Naros, G., Gharabaghi, A. Compensation or restoration: Closed-loop feedback of movement quality for assisted reach-to-grasp exercises with a multi-joint arm exoskeleton. Front Neurosci (2016) 10:280. doi: 10.3389/fnins.2016.00280.
4. Longo, D., Santini, G., Cherubini, G., Melchiorre, D., Ferrarello, F., Bagni, M.A. The muscle shortening maneuver in individuals with stroke: a consideration-of-concept randomized pilot trial. Top Stroke Rehabil (2022) 1–13. doi: 10.1080/10749357.2022.2145741.
5. Petrarca, M., Rossi, S., Bollea, L., Cappa, P., Castelli, E. Patient-centered rehabilitation, three years of gait recovery in a child affected by hemiplegia: case report. Eur J Phys Rehabil Med (2011) 47(1):35-47.
6. Pollock, C.L., Boyd, L.A., Hunt, M.A., Garland, S.J. Use of the Challenge Point Framework to Guide Motor Learning of Stepping Reactions for Improved Balance Control in People With Stroke: A Case Series Background and Purpose. Stepping reactions are important for walking (2014). doi: 10.2522/ptj.20130046.
7. Reinkensmeyer, D.J., Maier, M.A., Guigon, E., Chan, V., Akoner, O.M., Wolbrecht, E.T., et al. Do robotic and non-robotic arm movement training drive motor recovery after stroke, by a common neural mechanism? experimental evidence and a computational model. In: 2009 Annual International Conference of the IEEE Engineering in Medicine and Biology Society. IEEE (2009). p. 2439–41. doi: 10.1109/IEMBS.2009.5335353.
8. Rowe, J.B., Chan, V., Ingemanson, M.L., Cramer, S.C., Wolbrecht, E.T., Reinkensmeyer, D.J. Robotic Assistance for Training Finger Movement Using a Hebbian Model: A Randomized Controlled Trial. Neurorehabil Neural Repair (2017) 31(8):769–80. doi: 10.1177/1545968317721975.
9. Shaphe, A., Shalla, I.H., Al Baradie, R.S., Qasheesh, M. Efficacy of closed loop feedback system with augmented virtual reality visual cues training on gait and functional performance in stroke patients. Biosci Biotechnol Res Commun (2018) 11(1):70–5. doi: 10.21786/bbrc/11.1/10.
10. Vilimovsky, T., Chen, P., Hoidekrova, K., Petioky, J., Harsa, P. Prism adaptation treatment to address spatial neglect in an intensive rehabilitation program: A randomized pilot and feasibility trial. PLoS One. 2021 Jan 1;16. doi: 10.1371/journal.pone.0245425.
11. De Bruyn, N., Saenen, L., Thijs, L., Van Gils, A., Ceulemans, E., Essers, B., et al. Brain connectivity alterations after additional sensorimotor or motor therapy for the upper limb in the early-phase post stroke: A randomized controlled trial. Brain Commun (2021) 3(2). doi: 10.1093/braincomms/fcab074.
12. Doost, M.Y., Herman, B., Denis, A., Sapin, J., Galinski, D., Riga, A., et al. Bimanual motor skill learning and robotic assistance for chronic hemiparetic stroke: A randomized controlled trial. Neural Regen Res (2021) 16(8):1566–73. doi: 10.4103/1673-5374.301030.
13. García-Ramos, B.R., Villarroel, R., González-Mora, J.L., Revert, C., Modroño, C. Neurofunctional correlates of a neurorehabilitation system based on eye movements in chronic stroke impairment levels: A pilot study. Brain and behavior (2023) 13(8), doi: 10.1002/brb3.3049.
14. Gilmore, P.E., Spaulding, S.J. Motor learning and the use of videotape feedback after stroke. Top Stroke Rehabil (2007) 14(5):28–36. doi: 10.1310/tsr1405-28.
15. Hegazy, R.M., Alkhateeb, A.M., Abdelmohsen, A.M. Impact of a virtual reality program on post-stroke upper limb function: a randomized controlled trial. Physiotherapy Quarterly 2022; 30(4):81-86. doi:10.5114/pq.2021.111210
16. Huang, C.Y., Chiang, W.C., Yeh, Y.C., Fan, S.C., Yang, W.H., Kuo, H.C., et al. Effects of virtual reality-based motor control training on inflammation, oxidative stress, neuroplasticity and upper limb motor function in patients with chronic stroke: a randomized controlled trial. BMC Neurol (2022) 22(1). doi: 10.1186/s12883-021-02547-4.
17. Jonsdottir, J., Cattaneo, D., Recalcati, M., Regola, A., Rabuffetti, M., Ferrarin, M., et al. Task-oriented biofeedback to improve gait in individuals with chronic stroke: Motor learning approach. Neurorehabil Neural Repair (2010) 24(5):478–85. doi: 10.1177/1545968309355986.
18. Jonsdottir, J., Cattaneo, D., Regola, A., Crippa, A., Recalcati, M., Rabuffetti, M., et al. Concepts of motor learning applied to a rehabilitation protocol using biofeedback to improve gait in a chronic stroke patient: An A-B system study with multiple gait analyses. Neurorehabil Neural Repair (2007) 21(2):190–4. doi: 10.1177/1545968306290823.
19. Junata, M., Cheng, K.C.C., Man, H.S., Lai, C.W.K., Soo, Y.O.Y., Tong, R.K.Y. Kinect-based rapid movement training to improve balance recovery for stroke fall prevention: a randomized controlled trial. J Neuroeng Rehabil (2021) 18(1):150. doi: 10.1186/s12984-021-00922-3.
20. Kamatchi, K., Jibi, P., Jagatheesan, A., Harikrishnan, N. The Impact of Virtual Reality on Upper Extremity Function In Stroke Patients: A Pilot Study. Journal of Population Therapeutics and Clinical Pharmacology (2023) 30(8), 312–320. doi: 10.47750/jptcp.2023.30.08.033.
21. Kim, H., Kim, J., Jo, S., Lee, K., Kim, J., Song, C. Video augmented mirror therapy for upper extremity rehabilitation after stroke: a randomized controlled trial. Journal of neurology (2023) 270(2), 831–842. doi: 10.1007/s00415-022-11410-6.
22. Kim, G.J., Chen, P. Role of instruction adherence during highly structured robotic arm training on motor outcomes for individuals after chronic stroke. Am J Phys Med Rehabil (2020) 99(4):353–6. doi: 10.1097/PHM.0000000000001333.
23. Krishnamoorthy, V., Hsu, W.L., Kesar, T.M., Benoit, D.L., Banala, S.K., Perumal, R., et al. Gait training after stroke: A pilot study combining a gravity-balanced orthosis, functional lectrical stimulation, and visual feedback. Journal of Neurologic Physical Therapy (2008) 32(4):192–202. doi: 10.1097/NPT.0b013e31818e8fc2.
24. Longatelli, V., Pedrocchi, A., Guanziroli, E., Molteni, F., Gandolla, M. Robotic Exoskeleton Gait Training in Stroke: An Electromyography-Based Evaluation. Front Neurorobot (2021) 15. doi: 10.3389/fnbot.2021.733738.
25. Luque-Moreno, C., Cano-Bravo, F., Kiper, P., Solís-Marcos, I., Moral-Munoz, J.A., Agostini, M., et al. Reinforced Feedback in Virtual Environment for Plantar Flexor Poststroke Spasticity Reduction and Gait Function Improvement (2019). doi: 10.1155/2019/6295263.
26. Maenza, C., Wagstaff, D.A., Varghese, R., Winstein, C., Good, D.C., Sainburg, R.L. Remedial Training of the Less-Impaired Arm in Chronic Stroke Survivors With Moderate to Severe Upper-Extremity Paresis Improves Functional Independence: A Pilot Study. Front Hum Neurosci (2021) 15. doi: 10.3389/fnhum.2021.645714.
27. Maggio, M.G., Naro, A., Manuli, A., Maresca, G., Balletta, T., Latella, D., et al. Effects of Robotic Neurorehabilitation on Body Representation in Individuals with Stroke: A Preliminary Study Focusing on an EEG-Based Approach. Brain Topogr (2021) 34(3):348–62. doi: 10.1007/s10548-021-00825-5.
28. Mazzoleni, S., Tran V.D., Dario, P., Posteraro, F. Effects of Transcranial Direct Current Stimulation (tDCS) Combined With Wrist Robot-Assisted Rehabilitation on Motor Recovery in Subacute Stroke Patients: A Randomized Controlled Trial. IEEE Transactions on Neural Systems and Rehabilitation Engineering (2019) 27(7):1458–66. doi: 10.1109/TNSRE.2019.2920576.
29. Castro-Medina, K.G. Effects of Visual Feedback on Walking Speed for Stroke Patients: Single-case Design. Revista de investigación e innovación en ciencias de la salud (2023) 5(1):127-142. doi: https://doi.org/10.46634/riics.153.
30. Paolucci, T., Agostini, F., Mangone, M., Bernetti, A., Pezzi, L., Liotti, V., et al. Robotic rehabilitation for end-effector device and botulinum toxin in upper limb rehabilitation in chronic post-stroke patients: an integrated rehabilitative approach. Neurol Sci (2021) 42(12):5219-5229. doi: 10.1007/s10072-021-05185-3.
31. Park, M., Ko, M.H., Oh, S.W., Lee, J.Y., Ham, Y., Yi, H., et al. Effects of virtual reality-based planar motion exercises on upper extremity function, range of motion, and health-related quality of life: A multicenter, single-blinded, randomized, controlled pilot study. J Neuroeng Rehabil (2019) 16(1). doi: 10.1186/s12984-019-0595-8.
32. Piron, L., Turolla, A., Agostini, M., Zucconi, C.S., Ventura, L., Tonin, P., et al. Motor learning principles for rehabilitation: A pilot randomized controlled study in poststroke patients. Neurorehabil Neural Repair (2010) 24(6):501–8. doi: 10.1177/1545968310362672.
33. Powers, J., Wallace, A., Mansfield, A., Mochizuki, G., Patterson, K.K. The effect of frequency of feedback on overground temporal gait asymmetry post stroke. Top Stroke Rehabil (2022) 29(6):401–10. doi: 10.1080/10749357.2021.1943796
34. Pundik, S., McCabe, J., Skelly, M., Salameh, A., Naft, J., Chen, Z., et al. Myoelectric Arm Orthosis in Motor Learning-Based Therapy for Chronic Deficits After Stroke and Traumatic Brain Injury. Front Neurol (2022) 13. doi: 10.3389/fneur.2022.791144.
35. Sainburg, R.L., Maenza, C., Winstein, C., Good, D. Motor lateralization provides a foundation for predicting and treating non-paretic arm motor deficits in stroke. In: Advances in Experimental Medicine and Biology. Springer New York LLC (2016). p. 257–72. doi: 10.1007/978-3-319-47313-0_14.
36. Salameh, A., McCabe, J., Skelly, M., Duncan, K.R., Chen, Z., Tatsuoka, C., et al. Stance Phase Gait Training Post Stroke Using Simultaneous Transcranial Direct Current Stimulation and Motor Learning-Based Virtual Reality-Assisted Therapy: Protocol Development and Initial Testing. Brain Sci (2022) 12(6):701. doi: 10.3390/brainsci12060701.
37. Saleh, S., Fluet, G., Qiu, Q., Merians, A., Adamovich, S.V., Tunik, E. Neural Patterns of reorganization after intensive robot-assisted Virtual reality Therapy and repetitive Task Practice in Patients with chronic stroke. Front Neurol (2017) 8. doi: 10.3389/fneur.2017.00452.
38. Schreiber, J., Sober, L., Banta, L., Glassbrenner, L., Haman, J., Mistry, N., et al. Application of motor learning principles with stroke survivors. Occup Ther Health Care (2001) 13(1):23–44. doi: 10.1080/J003v13n01_03.
39. Smedes, F., Giacometti da Silva, L. Motor learning with the PNF-concept, an alternative to constrained induced movement therapy in a patient after a stroke; a case report. J Bodyw Mov Ther (2019) 23(3):622–7. doi: 10.1016/j.jbmt.2018.05.003.
40. Tretriluxana, J., Runnarong, N., Tretriluxana, S., Prayoonwiwat, N., Vachalathiti, R., Winstein, C. Feasibility investigation of the accelerated skill acquisition program (ASAP): Insights into reach-to-grasp coordination of individuals with postacute stroke. Top Stroke Rehabil (2013) 20(2):151–60. doi: 10.1310/tsr2002-151.
41. Tsaih, P.L., Chiu, M.J., Luh, J.J., Yang, Y.R., Lin, J.J., Hu, M.H. Practice variability combined with task-oriented electromyographic biofeedback enhances strength and balance in people with chronic stroke. Behavioural Neurology (2018) 2018. doi: 10.1155/2018/7080218.
42. Turolla, A., Daud Albasini, O.A., Oboe, R., Agostini, M., Tonin, P., Paolucci, S., et al. Haptic-based neurorehabilitation in poststroke patients: A feasibility prospective multicentre trial for robotics hand rehabilitation. Comput Math Methods Med (2013). doi: 10.1155/2013/895492.
43. van Vugt, F.T., Kafczyk, T., Kuhn, W., Rollnik, J.D., Tillmann, B., Altenmüller, E. The role of auditory feedback in music-supported stroke rehabilitation: A single-blinded randomised controlled intervention. Restor Neurol Neurosci (2016);34(2):297–311. doi: 10.3233/RNN-150588.
44. Winstein, C., Kim, B., Kim, S., Martinez, C., Schweighofer, N. Dosage Matters. Stroke (2019) 50(7), 1831–1837. doi: 10.1161/STROKEAHA.118.023603.
45. Zollo, L., Rossini, L., Bravi, M., Magrone, G., Sterzi, S., Guglielmelli, E. Quantitative evaluation of upper-limb motor control in robot-aided rehabilitation. Med Biol Eng Comput (2011) 49(10):1131–44. doi: 10.1007/s11517-011-0808-1.
